# Peripheral tissues reprogram CD8^+^ T cells for pathogenicity during graft-versus-host disease

**DOI:** 10.1172/jci.insight.97011

**Published:** 2018-03-08

**Authors:** Pedro Santos e Sousa, Séverine Ciré, Thomas Conlan, Laura Jardine, Claire Tkacz, Ivana R. Ferrer, Cara Lomas, Sophie Ward, Heather West, Simone Dertschnig, Sven Blobner, Terry K. Means, Stephen Henderson, Daniel H. Kaplan, Matthew Collin, Vincent Plagnol, Clare L. Bennett, Ronjon Chakraverty

**Affiliations:** 1 Haematology, UCL Cancer Institute and Institute of Immunity & Transplantation, London, United Kingdom (UK).; 2 Institute of Cellular Medicine, Newcastle University, Newcastle upon Tyne, UK.; 3 UCL Genetics Institute, London, UK.; 4 Center for Immunology and Inflammatory Diseases, Massachusetts General Hospital, Charlestown, Massachusetts, USA.; 5 Bill Lyons Informatics Centre, UCL Cancer Institute, London, UK.; 6 Department of Immunology, University of Pittsburgh, Pittsburgh, Pennsylvania, USA.

**Keywords:** Immunology, Transplantation, Molecular pathology, Stem cell transplantation, T cell development

## Abstract

Graft-versus-host disease (GVHD) is a life-threatening complication of allogeneic stem cell transplantation induced by the influx of donor-derived effector T cells (T_E_) into peripheral tissues. Current treatment strategies rely on targeting systemic T cells; however, the precise location and nature of instructions that program T_E_ to become pathogenic and trigger injury are unknown. We therefore used weighted gene coexpression network analysis to construct an unbiased spatial map of T_E_ differentiation during the evolution of GVHD and identified wide variation in effector programs in mice and humans according to location. Idiosyncrasy of effector programming in affected organs did not result from variation in T cell receptor repertoire or the selection of optimally activated T_E_. Instead, T_E_ were reprogrammed by tissue-autonomous mechanisms in target organs for site-specific proinflammatory functions that were highly divergent from those primed in lymph nodes. In the skin, we combined the correlation-based network with a module-based differential expression analysis and showed that Langerhans cells provided in situ instructions for a Notch-dependent T cell gene cluster critical for triggering local injury. Thus, the principal determinant of T_E_ pathogenicity in GVHD is the final destination, highlighting the need for target organ–specific approaches to block immunopathology while avoiding global immune suppression.

## Abstract 2

During the evolution of graft-versus-host disease, effector CD8+ T cells are re-programmed by tissue-autonomous mechanisms to develop idiosyncratic pathogenic functions in target organs.

## Introduction

Acute graft-versus-host disease (GVHD) occurs as a result of a tissue-tropic, pathogenic immune response orchestrated by donor T cells following allogeneic hematopoietic stem cell transplantation (1–3). Tissue inflammation frequently emerges despite the concurrent use of immune suppressive agents targeting systemic T cells; early treatment resistance is common and associated with a high risk of mortality (4). Although proinflammatory immune signatures from blood can predict patients likely to develop breakthrough or treatment-resistant acute GVHD (5–7), it is currently unclear whether earlier interventions can change the disease course. There is therefore an unmet need to identify targetable pathways that are critical to the initiation and propagation of tissue injury.

Following experimental bone marrow transplantation (BMT), allogeneic T cells undergo an initial 3- to 4-day phase of activation and proliferation in recipient secondary lymphoid organs (SLOs), before exit into the blood and subsequent trafficking to peripheral tissues where they are first detectable at day 6 or 7 (8). Fate mapping of allogeneic T cells in GVHD suggests that early differentiation programs of effector T cells (T_E_) are highly plastic leading to a high level of heterogeneity at a population level (9). Such diversity could potentially arise through either stochastic or instructional mechanisms (10), the latter reflecting responsiveness to environmental cues. In the latter case, early effector programs could be subject to modification following exposure to variations in the strength or duration of antigenic stimulation, costimulation, cytokines, or help (11). Although most studies have focused on how such instructions impact on early effector programs in SLOs, T_E_ will also be subject to a distinct repertoire of signals following their recruitment to nonlymphoid tissues. Indeed, early effector programs of T_E_ entering peripheral tissues affected by GVHD are still highly plastic and can be reset under conditions where they recirculate to lymph nodes (LNs) (12). Furthermore, recent studies in healthy volunteers have revealed unexpected diversity in the phenotypic and functional properties of T cells isolated from peripheral tissues compared with blood or LNs (13), suggesting that effector programs initiated in lymphoid organs can be overwritten when T cells are recruited to other sites. Although dynamic interactions in tissues regulate effector responses to commensal flora or are required for specialized memory differentiation (14–16), the extent to which peripheral tissues directly reprogram T cells for pathogenicity has not been explored in GVHD.

To investigate the role of GVHD target organs in shaping pathogenic T cell function, we therefore used a network biological approach in order to construct an unbiased spatial map of effector CD8^+^ T_E_ differentiation at multiple locations during the evolution of GVHD. We found that murine and human T_E_ are reprogrammed in nonlymphoid tissues for site-specific, proinflammatory functions that are highly divergent from those triggered in lymphoid organs, as well as from each other. In the skin, epidermal Langerhans cells (LCs) are required for the upregulation of a Notch-dependent T cell gene cluster that is critical for local pathogenicity. Our data therefore demonstrate the tissue-autonomous programming of pathogenic T_E_ in GVHD, and suggest the need for precision targeting of immune pathological processes that are specific to target organs.

## Results

### TCR repertoire–independent and tissue-autonomous divergence of T cell effector programs in lymphoid and nonlymphoid organs.

**Figure 1:**
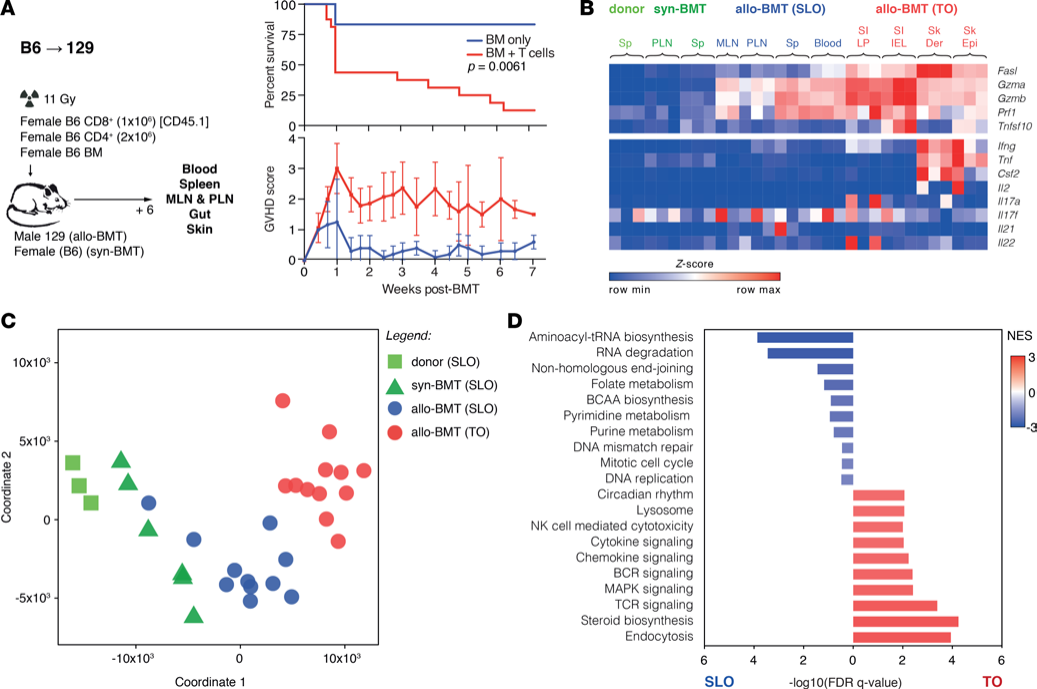
T_E_ gene expression profiles are functionally and spatially divergent. (**A**) Left: Experimental setup of the B6→129 BMT model. Right, top graph: Kaplan-Meier survival curve (log-rank Mantel-Cox test). Right, bottom graph: clinical GVHD score over time (mean ± SD). BM only (*n* = 6), BM + T cells (*n* = 16). (**B**) Heatmap showing SLO- and GVHD target organ–derived T_E_ expression of cytotoxic and cytokine genes known to be important in T_E_ differentiation. (**C**) MDS plot showing the proximity of the transcriptional profiles of donor-derived CD8^+^ T cells isolated from different organs. (**D**) Graph showing the FDR *q* value (bars) and NES (color code) calculated by GSEA, comparing the top 10 enriched KEGG pathways in allo-BMT SLO (blue) and GVHD TO (red) groups. BCAA, branched-chain amino acid; BM, bone marrow; BMT, BM transplantation; Der, dermis; Epi, epidermis; FDR, false discovery rate; GSEA, gene set enrichment analysis; GVHD, graft-versus-host disease; IEL, intraepithelial lymphocyte; LP, lamina propria; MDS, multidimensional scaling; MLN, mesenteric lymph node; NES, normalized enrichment score; PLN, peripheral lymph node; SI, small intestine; SLO, secondary lymphoid organ; Sk, skin; T_E_, effector T cell; TO, target organ.

In initial experiments, we characterized the transcriptional response of donor CD8^+^ T_E_ as they trafficked to multiple sites during the evolution of GVHD. Using a clinically relevant model of H-2^b^ MHC-matched, multiple minor antigen–mismatched BMT (B6→129) involving transfer of polyclonal donor CD4^+^ and CD45.1^+^ CD8^+^ T cells, we flow sorted to high purity CD45.1^+^ CD8^+^ T_E_ from individual SLOs (blood, spleen, mesenteric/peripheral LNs) and GVHD target organs (skin dermis and epidermis, small intestinal lamina propria [LP] and intraepithelial lymphocyte [IEL] compartments, liver) on day 6 following transplant. Naive T cells, and donor CD8^+^ T cells undergoing lymphopenia-induced proliferation in SLOs from syngeneic BMT recipients, served as controls (Figure 1A). We obtained a total of 36 samples (3 replicate samples/tissue from 3 independent experiments, pooling where necessary from multiple mice from individual experiments), with a median CD8^+^ T cell purity of 98.7% (range 97.3%–99.7%) and median cell number/sample of 4.1 × 10^4^ (range 0.7 × 10^4^ to 15.3 × 10^4^). To determine how gene expression profiles of T_E_ isolated from individual SLOs versus GVHD target organs compared, we initially evaluated the expression of cytotoxic and cytokine molecules known to be important in T_E_ differentiation. As shown in Figure 1B, this analysis showed significant variation between SLOs and GVHD target organs, and between target organs; for example, the cytotoxic genes *Gzma* and *Gzmb* were most highly expressed by gut T_E_, whereas a subset of proinflammatory cytokine genes (e.g., *Tnf*, *Ifng*, and *Csf2*) were mainly expressed by skin T_E_. In order to obtain an overview for the relationship between individual samples according to location, we next performed multidimensional scaling (MDS) following subtraction of skin and gut tissue-specific genes from the entire data set to correct for possible artifacts created by highly expressed genes by the small minority (median 1.3%, range 0.3%–2.7%) of contaminating non–T cells (17) (Figure 1C; for comparison, the MDS plot without subtraction is shown in Supplemental Figure 1; supplemental material available online with this article; https://doi.org/10.1172/jci.insight.97011DS1). As expected, T_E_ from allogeneic recipients segregated independently from both naive T cells and those undergoing lymphopenia-induced proliferation. However, we also observed a clear separation between T_E_ derived from SLOs and GVHD target organs in allogeneic BMT recipients (Figure 1C). To further determine which molecular pathways were associated with T_E_ isolated from SLOs versus GVHD target organs, we performed gene set enrichment analysis (GSEA). As shown in Figure 1D, SLO T_E_ showed enrichment for biological processes relating to proliferative and metabolic fitness, for example cell cycle, RNA processing, and DNA replication, whereas GVHD target organ T_E_ showed enrichment for proinflammatory pathways, for example MAP kinase signaling, TCR signaling, chemokine-chemokine receptor or cytokine-cytokine receptor interactions.

**Figure 2:**
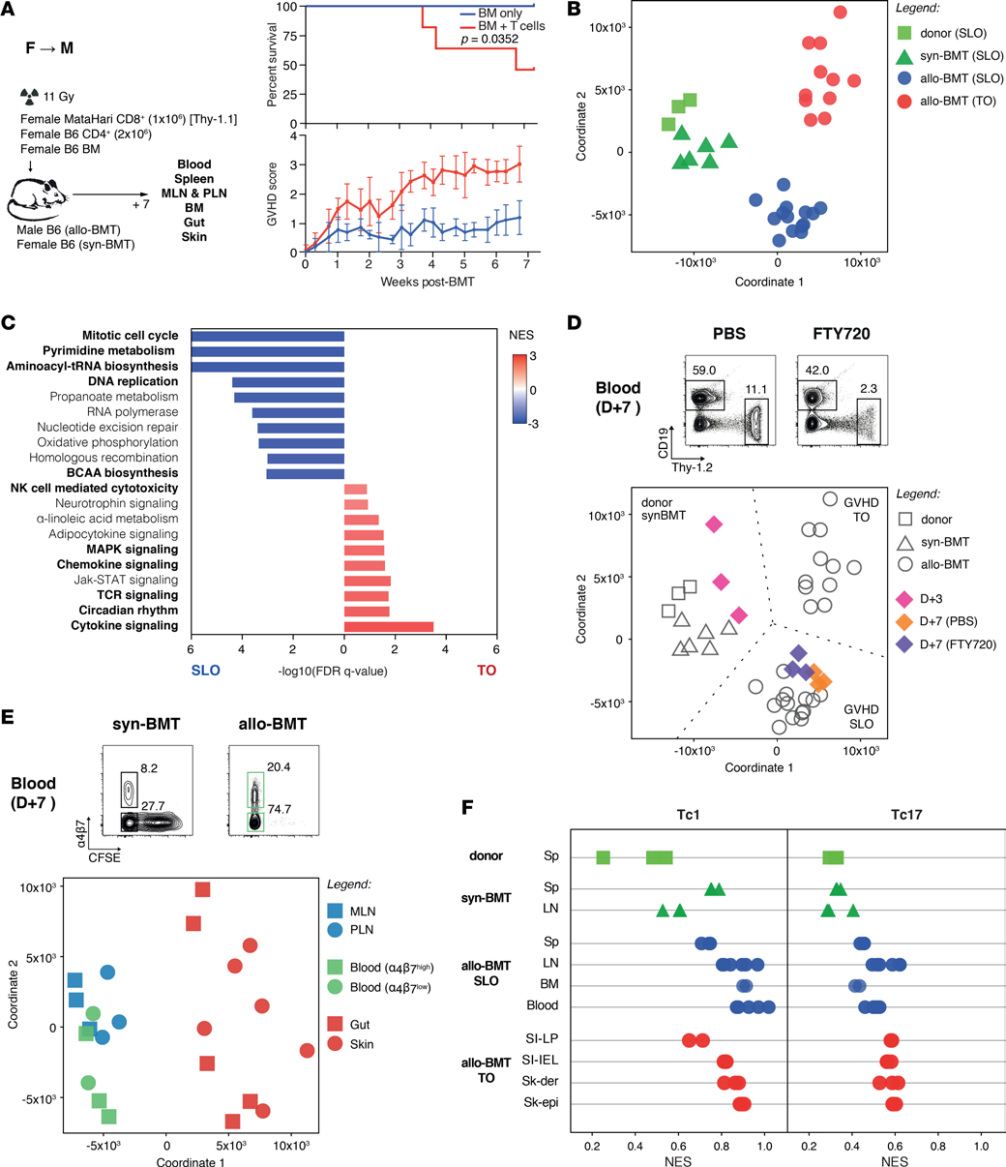
T_E_ effector program diversity in SLO and peripheral tissues is tissue autonomous and TCR repertoire independent. (**A**) Left: Experimental setup of the F→M BMT model. Right, top graph: Kaplan-Meier survival curve (log-rank Mantel-Cox test). Right, bottom graph: clinical GVHD score over time (mean ± SD). BM only (*n* = 6), BM + T cells (*n* = 11). (**B**) MDS plot showing the proximity of transcriptional profiles of donor-derived CD8^+^ T cells isolated from different organs. (**C**) Graph showing the FDR *q* value (bars) and NES (color code) calculated by GSEA, comparing the top 10 enriched KEGG pathways in allo-BMT SLO (blue) and GVHD TO (red) groups. Pathways in common with the B6→129 BMT model are highlighted in bold. (**D**) F→M BMT + T cell recipients were treated with daily intraperitoneal FTY720 or PBS from day 3 onwards (*n* = 3, each group). Top: Representative plots of whole-blood staining for Thy-1.2 and CD19 at day 7 after transplant in FTY720- and PBS-treated BMT recipients. Bottom: MDS plot showing the proximity of transcriptional profiles of naive input T cells and donor-derived CD8^+^ T cells from the LNs at day 3, and at day 7 with or without FTY720-mediated prevention of T_E_ egress to the periphery. (**E**) Top: sorting strategy of peripheral blood T cells (right panel; green gates). Bottom: MDS plot showing similarity of the transcriptional profiles of donor-derived CD8^+^ T cells isolated from the GVHD target organs (gut and skin) and from their respective draining LNs, and GVHD target organ–tropic peripheral blood subsets. (**F**) Representation of Tc1 and Tc17 gene signature expression in each sample, evaluated by single-sample GSEA. BCAA, branched-chain amino acid; BM, bone marrow; BMT, BM transplantation; Der, dermis; D+, number of days after BMT; Epi, epidermis; FDR, false discovery rate; GSEA, gene set enrichment analysis; GVHD, graft-versus-host disease; IEL, intraepithelial lymphocyte; LN, lymph node; LP, lamina propria; MDS, multidimensional scaling; MLN, mesenteric LN; NES, normalized enrichment score; PLN, peripheral LN; SI, small intestine; SLO, secondary lymphoid organ; Sk, skin; T_E_, effector T cell; TO, target organ.

Sampling of T cells from the peripheral blood and target tissues in human patients with GVHD has suggested that TCR repertoires at the respective sites are frequently distinct (18, 19). We therefore reasoned that differences in gene expression in T_E_ from SLOs and GVHD target organs could occur (a) because of differential selection of preexisting variants from the bulk T cell repertoire or (b) because of tissue environment–dependent reprogramming, a process that would be expected to be independent of the TCR repertoire. To exclude the former possibility that the observed differences in T_E_ gene expression between the SLOs and GVHD target organs related to preexisting variation in TCR repertoire (for example, due to selective expansion of atypical T_E_ clones recognizing antigens expressed uniquely in one set of tissues), we repeated these experiments in an additional B6 female → B6 male (F→M) BMT model involving transfer of naive MataHari CD8^+^ T cells transgenic for a TCR that recognizes a single, ubiquitous HY antigen, D^b^‑Uty (20, 21) (Figure 2A). In this model, the TCR repertoire is fixed and therefore differences in gene expression between SLO and target organ T_E_ will be independent of differences in TCR repertoire. Using the same approach as the B6→129 model but including additional T_E_ from the bone marrow (BM), we obtained a total of 42 samples from GVHD mice and syngeneic F→F BMT controls (3 replicate samples/tissue from 3 independent experiments, pooling where necessary from multiple mice from individual experiments), with a median CD8^+^ T cell purity of 98.6% (range 95.2%–98.8%) and median cell number/sample of 4.1 × 10^4^ (range 0.4 × 10^4^ to 25 × 10^4^). Again, we found that MataHari T_E_ profiles from SLOs and GVHD target organs segregated separately by MDS (Figure 2B) and GSEA showed similar enrichment for proliferative programs in SLO T_E_ versus proinflammatory functions in GVHD target organ T_E_, as we had observed in the B6→129 model (Figure 2C; bold text showing the programs that overlap between the 2 models). A high degree of overlap between the T_E_ profiles from each tissue in the F→M and B6→129 data sets was also observed using a correlation matrix as shown in Supplemental Figure 2A. Together, these data indicate that the major differences in T_E_ profiles between SLOs and GVHD target organs emerge through mechanisms that are independent of the TCR repertoire. We also considered a second possibility that the distinct gene expression profiles of T_E_ from GVHD target organs was stochastic and due to the selective enrichment of T_E_ that had undergone optimal activation before rapid homing to peripheral tissues. Thus, we devised an experiment whereby T_E_ could be constrained to remain in the LNs following activation and before analysis of gene expression; if the divergence in gene signatures between LNs and target tissues was time dependent but tissue independent, we would expect that T_E_ trapped in LNs would display a more tissue-specific transcription profile. Thus, we repeated the F→M experiments, but treated a cohort of animals from day 3 (a time point before MataHari T cells are detectable in blood or GVHD organs; data not shown) with FTY720 (a sphingosine-1-phosphate antagonist) to trap T_E_ within the LNs. As shown in Figure 2D, T_E_ transcriptional profiles in LNs on day 3 were in fact closer to the profiles of naive input T cells, whereas the T_E_ differentiation profile on day 7 in FTY720-treated mice was almost identical to controls, without any skewing towards a tissue-like signature. These findings therefore demonstrate that differences in gene expression between T_E_ of SLOs versus GVHD target organs were not related to the selection of optimally activated T_E_, but instead were intimately related to tissue location. Finally, because dendritic cells (DCs) in the draining LNs can program selective homing of T_E_ to the gut or skin (22), we also considered whether tissue-specific reprogramming could be traced back to local imprinting of T_E_ outside the target organs. However, the transcriptional profiles of CFSE^lo^α_4_β_7_^hi^ blood T_E_ (i.e., cells that have proliferated following transfer of labeled cells and express a gut-homing phenotype) and gut-draining mesenteric LN T_E_ were more similar to CFSE^lo^α_4_β_7_^lo^ blood T_E_ and skin-draining peripheral LN T_E_ than they were to the profiles of T_E_ isolated directly from the gut LP or IEL compartments (Figure 2E). Taken together, these data reveal the critical role of peripheral tissues in directly instructing the programs adopted by T_E_ recruited to these sites.

We next asked to what extent previously published transcriptional Tc1 (23) and Tc17 (9) signatures were enriched in T_E_ signatures isolated from the individual organs in our experiments. We therefore applied single-sample GSEA (24), a method that tests for enrichment of individual gene sets by absolute expression rather than by comparison with another sample. We found that both Tc1 and Tc17 signatures were enriched across both SLO- and target organ–derived T_E_ in GVHD mice (Figure 2F). Taken together with Figure 1, these data identify distinct differentiation states of T_E_ in GVHD corresponding to proliferation in SLOs versus deployment of proinflammatory functions in target organs. In contrast, published signatures for Tc1 or Tc17 populations do not segregate according to cell position in the host.

### Gene correlation network analysis in GVHD.

**Figure 3:**
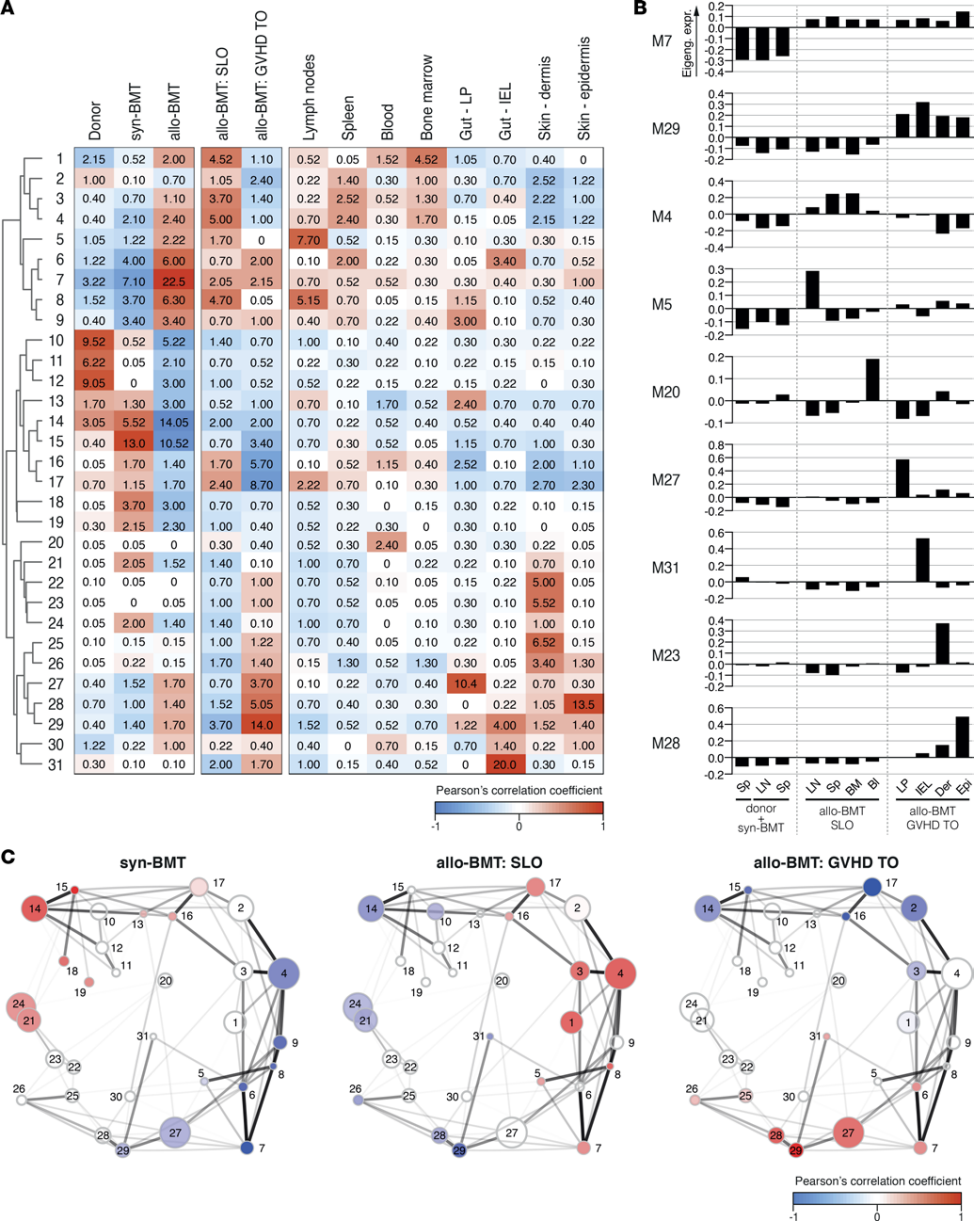
Donor T_E_ transcription conforms to a spatially diverse modular architecture. (**A**) Correlation matrix depicting the association between individual gene modules defined by weighted gene coexpression network analysis–defined modules and the experimental groups (donor, syn-BMT, allo-BMT), GVHD subgroups (SLO, TO), and GVHD individual organs. Cell color and cell number indicate Pearson’s correlation coefficient and corresponding –log10(*P* value), respectively. (**B**) Bar graphs showing the mean eigengene expression in each of the tissues, for pan-allo-BMT (M7), pan-GVHD TO (M29), SLO-selective (M4), and tissue-specific modules (M5, M20, M23, M27, M28, M31). (**C**) Eigengene network constructed with Cytoscape, in which the nodes (circles) represent the gene modules (circle area proportional to the number of genes in the module) and the edges (lines) represent the correlation between each pair of modules (line thickness proportional to Pearson’s correlation coefficient; line transparency proportional to *P* value). The nodes are spatially arranged according to the adjacency between gene modules and the color of the nodes reflects the correlation between the modules and the group of tissues represented. Bl, blood; BM, bone marrow; BMT, BM transplantation; Der, dermis; Epi, epidermis; GVHD, graft-versus-host disease; IEL, intraepithelial lymphocyte; LN, lymph node; LP, lamina propria; SLO, secondary lymphoid organ; Sp, spleen; TO, target organ.

To better define the evolution of transcriptional programs of T_E_ according to their precise location, we therefore developed an unbiased analytical pipeline based on correlation network analysis and downstream validation (summarized in Supplemental Figure 3 and explained in following sections). We first constructed an unbiased spatial map employing weighted gene coexpression network analysis (WGCNA), an unsupervised method that clusters genes based on their expression profiles by pairwise correlations between all the genes, thus generating a biological network (25). This computational approach is based on the low probability that multiple transcripts will follow a complex pattern of expression across many conditions only by chance. Once this biological network has been built, WGCNA is able to identify groups of genes (termed ‘modules’), which show a coexpression pattern within the data set; such gene clusters may therefore constitute coherent and biologically meaningful transcriptional units. The modules can then be interrogated for associations with specific conditions (traits) within the data set, to determine module-to-trait correlations, which helps to find groups or conditions of specific or shared modules. Using the data set derived from the F→M BMT model and controls, WGCNA was applied to identify distinct modules of coexpressed genes that were then mapped to T_E_ from each individual tissue. By adopting this method, we were able to identify 31 distinct gene modules (each designated with the prefix ‘M’) whose expression covaried substantially according to the experimental condition (syngeneic or allogeneic BMT) and the tissue source of the T cells (Figure 3A). When analyzed according to individual module eigengene expression (that is the first principal component of a given module), we identified further patterns of module expression varying according to the experimental condition and position of T cells (Figure 3B). Some module eigengenes (e.g., M5 in LNs, M20 in blood, M27 in LP, M31 in IEL, M23 in dermis, and M28 in epidermis) were highly correlated with individual tissue subcompartments, whereas others were correlated with multiple SLOs (e.g., M4) or GVHD target organs (e.g., M29). Additionally, we identified a small group of modules (especially M7, but also M6, M8, and M9) that covaried with multiple tissues (both SLO and target organ) following allo-BMT, and we refer to these as “bridging modules” (Figure 3A; eigenegene expression for M7 shown in Figure 3B). To determine how each individual module correlated with other modules, we created an eigengene network map where individual module relationships could be visualized on the basis of proximity and intermodular connectivity, with thicker lines indicating higher levels of correlation (Figure 3C). As anticipated from the previous analyses depicted in Figure 2, SLO- and GVHD target organ–related modules segregated into 2 distinct groups, while the bridging modules (M6–M9) were highly correlated with each other and linked the 2 groups.

In order to determine whether the WGCNA-derived modules were robust and reproducible, we performed a module preservation analysis, a method that uses a permutation test to define a statistic (*Z*_sum_) that summarizes the evidence that the network topology of any given module in a data set is preserved in a completely independent data set (26). Indeed, 30 of the 31 gene clusters identified in the F→M BMT model were conserved in the B6→129 BMT model, as demonstrated by the individual composite preservation measurement, *Z*_sum_ > 2.0 (Supplemental Figure 2B). Furthermore, by applying the same WGCNA method to the B6→129 data set we identified 22 modules (MA–MV) with similar tissue expression patterns (Supplemental Figure 2, C and D). Using the same module preservation analysis, 22 of the 22 WGCNA modules derived from the B6→129 BMT model were conserved in the F→M BMT model (Supplemental Figure 2E).

For further analysis, we prioritized the 19 gene clusters from the F→M BMT model, which met a higher stringency cutoff for module preservation in the B6→129 BMT model (*Z*_sum_ ≥ 10) (26). To gain insight into the biological processes associated with each module, we classified them according to the overrepresented Gene Ontology (GO) categories, identified their putative driver genes (i.e., genes with the highest intramodular connectivity determined by WGCNA), and used iRegulon (27) to map the transcription factors (TFs) predicted to act as upstream regulators (summarized in Table 1; full list in Supplemental Table 1, A–C). Several strongly interconnected gene clusters segregated primarily with the SLO origin of T_E_ (Figure 3C). M17 was strongly correlated with T_E_ from the LNs but negatively correlated with T_E_ from GVHD target organs; this module was linked to Toll-like receptor and retinoic acid–inducible gene 1 signaling and accordingly, driver genes encoded proteins dictating responsiveness to type I interferons, e.g., *Irf9*. M1 mapped to blood and BM-derived T_E_ and contained genes related to cell cycle and DNA replication, e.g., *Aurkb* and *Cdk1*. M3 segregated with the spleen and BM T_E_ and contained genes that were almost exclusively related to fatty acid oxidation and oxidative phosphorylation, e.g., *Hadh*, *Cs*, and *Mdh1*. Of the bridging gene clusters mapping to both SLOs and GVHD target tissues (M6–M9), M7 was the most densely interconnected and contained multiple genes encoding proteins involved in cytoskeletal reorganization and transendothelial migration (e.g., *Anxa2*, *S100a4*, and *Adap1*). M29 segregated with the majority of GVHD target organs and can be considered a pan-GVHD target organ gene cluster; drivers included genes encoding receptors (e.g., *Tnfr2*), adaptors (e.g., *Traf1/4*, *Gadd45b*, and *Nr4a2*), and TFs that regulate Th/Tc1 and Th/Tc17 proinflammatory cytokine generation (e.g., *Rel*, *Fosl2*, *Kdm6b*, *Skil*, and *Chd7*). The largest module that correlated with GVHD target organs was M27, which segregated mainly with the LP; this cluster was enriched for multiple intracellular signaling gene pathways including MAPK (e.g., *Map3k1* and *Relb*) and JAK-STAT (e.g., *Jak2*, *Stat3*, and *Stat5a*). M28 was highly correlated with epidermal T_E_ and contained driver genes associated with Notch signaling (*Rbpj* and *Furin*), multiple proinflammatory cytokines (e.g., *Ifng*, *Il2*, *Il3*, *Il13*, *Il17a*, *Csf1*, and *Csf2*), cytokine receptors and downstream adaptors (e.g., *Il2ra*, *Il1r1*, and *Il18rap*). Together, the data in Figure 3 and Table 1 reveal a spatially diverse modular architecture for T_E_ differentiation in GVHD, with programs for pathogenicity being highly concentrated in the target organs.

### Blood- and skin-correlated T cell modules in experimental GVHD correlate with signatures identified in human patients.

**Figure 4:**
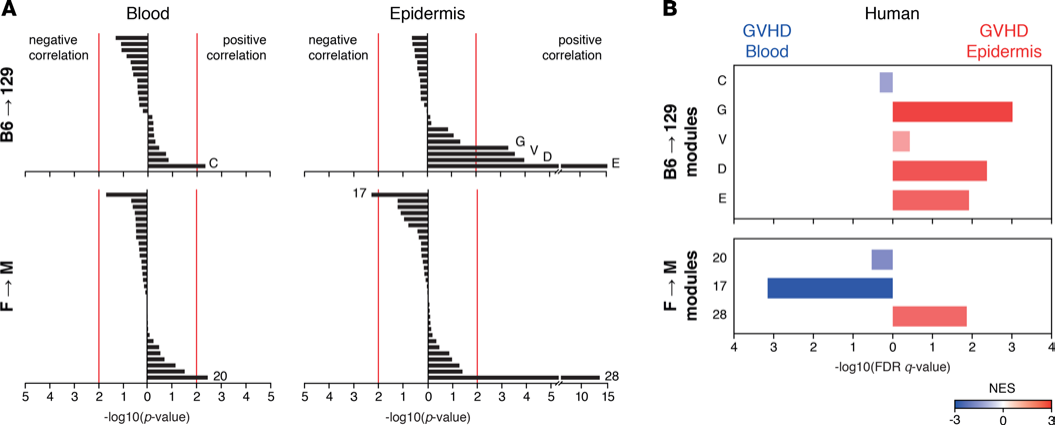
Blood- and skin-correlated T cell modules are also identifiable in human patients. (**A**) Bar graph showing the B6→129 and F→M WGCNA-defined modules ordered according to their correlation with the blood or the epidermis. Red line indicates *P* = 0.01. (**B**) Graph showing the FDR *q* value (bars) and NES (color code) calculated by GSEA, comparing the enrichment for the blood- and skin-correlated B6→129 and F→M WGCNA-defined modules in the blood (blue) and epidermis (red) samples of human patients at the onset of acute skin GVHD. FDR, false discovery rate; GSEA, gene set enrichment analysis; GVHD, graft-versus-host disease; NES, normalized enrichment score; SLO, secondary lymphoid organs; TO, target organs; WGCNA, weighted gene coexpression network analysis.

To test whether T_E_ gene modules correlating with target tissues that we identified in experimental GVHD would also be conserved at similar locations in human patients developing GVHD, we performed RNA sequencing (RNAseq) on CD8^+^ T cells obtained simultaneously from the blood and epidermis of 5 patients at the onset of acute pattern skin GVHD (Supplemental Table 2). We then ranked the murine modules derived from the F→M or B6→129 models according to their correlation with either the epidermis or blood, using a cutoff –log10(*P* value) > 2 to identify the modules with the greatest positive or negative correlation for each site (Figure 4A). As shown in Figure 4B, the epidermis- and blood-specific modules from each model segregated with the transcriptomes from human T_E_ from the respective sites, indicating interspecies conservation according to location.

### Entry of T_E_ into the epidermis is associated with activation of a Notch-dependent T cell program.

**Figure 5:**
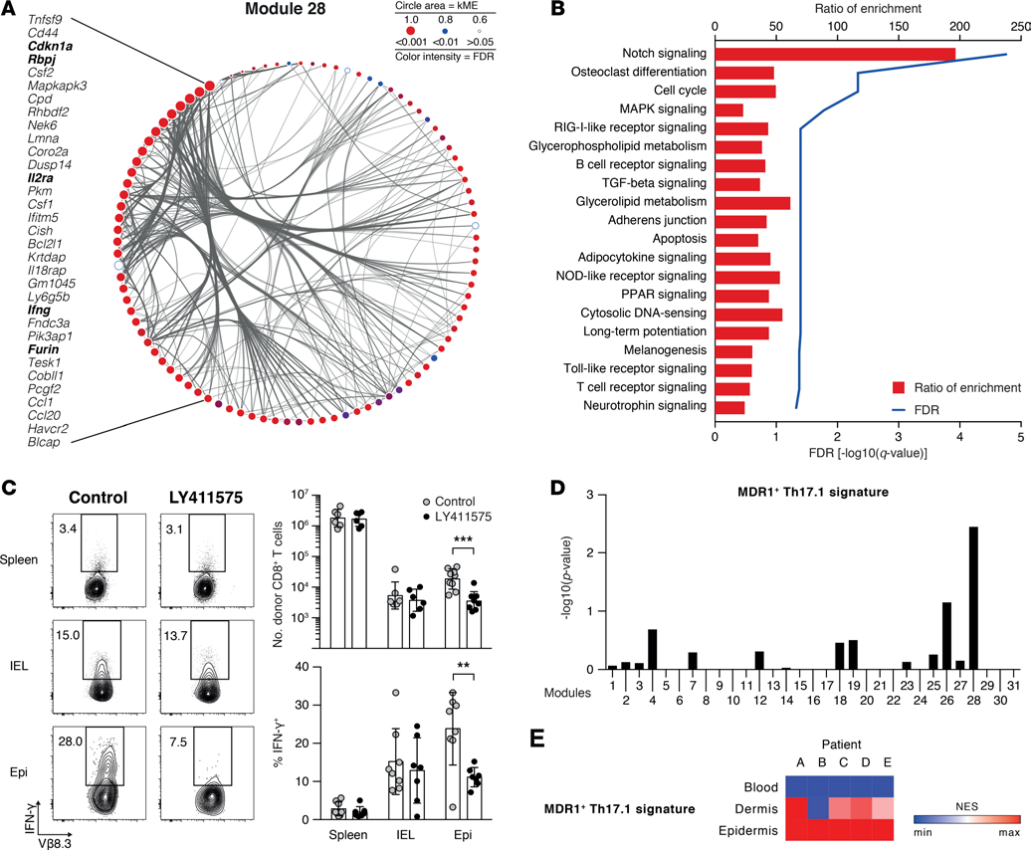
Notch signaling is a locale-specific regulator of T_E_ functions within the epidermis. (**A**) Cytoscape-generated visualization of the network connections among the 100 most connected genes in M28. Nodes represent the genes (circle area proportional to the intramodular connectivity, kME) and the color reflects the FDR *q* value of its correlation with the module; edges represent the topological overlap between genes (line thickness proportional to adjacency). Notch pathway–related genes and Notch downstream targets are highlighted. (**B**) Graph showing the ratio of enrichment (bars) and FDR *q* values (line) for pathways predicted by WebGestalt to regulate M28. (**C**) Effect of in vivo Notch signaling blockade upon alloreactive T_E_ tissue infiltration and effector function. F→M BMT recipients were treated on days 5 and 6 with LY411575 or vehicle i.p. On day 7, IFN-γ synthesis by MataHari T cells in the spleen, IEL, and epidermis (left: representative flow cytometric plots; bottom right: summary data) and corresponding numbers of MataHari T cells isolated from each site (top right: summary data) were determined. Data derived from 3 independent experiments: LY411575 *n* = 7, vehicle *n* = 8 (all graphs showing mean ± SD). **P ≤ 0.01, ***P ≤ 0.001 by ANOVA with Holm-Sidak correction for multiple comparisons. (**D**) Graph showing module association with resistance to immunosuppressive therapies assessed by determining the overrepresentation of gene signature specific for a human MDR1^+^ Th1/Th17 subset that is resistant to glucocorticoids. Hypergeometric test. (**E**) Heatmap showing the relative enrichment for the MDR1^+^ Th1/Th17 gene signature in blood, dermis, and epidermis samples from GVHD patients as determined by single-sample GSEA. BMT, bone marrow transplantation; Epi, epidermis; FDR, false discovery rate; GSEA, gene set enrichment analysis; GVHD, graft-versus-host disease; IEL, intraepithelial lymphocyte; kME, intramodular connectivity; NES, normalized enrichment score; SLO, secondary lymphoid organ; T_E_, effector T cell; TO, target organ.

We next sought to define how entry into a specific GVHD target organ would confer biological function to T_E_ at that site. Therefore, we focused on the putative pathogenic M28 gene cluster in the F→M BMT model (Figure 5A and Table 1), which was highly conserved in human patients with skin GVHD (Figure 4B) and uniquely correlated with murine T_E_ from the epidermis (Figure 3B). Prominent among the putative M28 driver genes were the Notch pathway–related genes *Rbpj* and *Furin* and Notch downstream targets (*Ifng*, *Il2ra*, and *Cdnk1a*) (Figure 5A). To determine the potential regulators of M28 gene expression we used Cytoscape’s iRegulon plugin that measures enrichment for TF binding sites in the putative regulatory regions of each gene and identifies TFs predicted to bind to these motifs (e.g., EP300, HDAC2, and HES5 were predicted TFs in this analysis). Functional enrichment analysis for the predicted TF was performed using WebGestalt (28) and strongly suggested Notch signaling as the main upstream pathway inducing the M28 genetic program (Figure 5B, ratio of enrichment = 196.6, FDR *q* value = 1.75 × 10^–5^). Of note, M28 also overlapped significantly with 2 epidermis-specific modules (MD and ME) identified in the B6→129 model (Supplemental Figure 4A; MD sharing 33 genes, *P* value = 1.67 × 10^–9^; ME sharing 49 genes, *P* value = 1.13 × 10^–17^, hypergeometric test). Using the same approach as for Figure 5B, Notch was also identified as a potential upstream regulator of both these modules (Supplemental Figure 4B; MD ratio of enrichment = 96.6, FDR *q* value = 0.002; ME ratio of enrichment = 36.7, FDR *q* value = 0.04).

To test whether Notch signaling would affect the numbers and functions of T_E_ following their recruitment to peripheral tissues in vivo, we therefore treated male BMT recipients with the γ‑secretase inhibitor LY411575 or vehicle on days 5 and 6, a time point that follows initial activation of T cells in SLOs and their subsequent entry into peripheral tissues. As shown in Figure 5C, delayed Notch inhibition reduced T_E_ accumulation and the generation of IFN-γ, a known Notch target (29) and M28 driver gene. In sharp contrast, Notch inhibition had no effect on T_E_ numbers or generation of IFN-γ in the spleen or the gut intraepithelial compartment. Thus, Notch signaling played a locale-specific role in regulating T_E_ functions within the epidermis.

Because Notch signaling can mediate a priori T cell resistance to glucocorticoids (GCs) (30), we explored the possibility that M28 would be enriched for genes associated with resistance to immunosuppressive therapies. Indeed, among all the WGCNA-defined modules, genes contained in the M28 cluster demonstrated the greatest degree of overlap with a gene signature specific for a human MDR1^+^ Th1/Th17 subset that is resistant to GCs (31) (Figure 5D). A similar overlap between the B6→129 module MD and the GC-resistance signature was also observed (Supplemental Figure 4C). This GC-resistance signature was also detectable from CD8^+^ T_E_ derived from the epidermis of human patients at the onset of acute GVHD (Figure 5E), suggesting the possibility that resistant populations may already be present at diagnosis.

### LCs are required for T_E_ pathogenicity in the epidermis.

**Figure 6:**
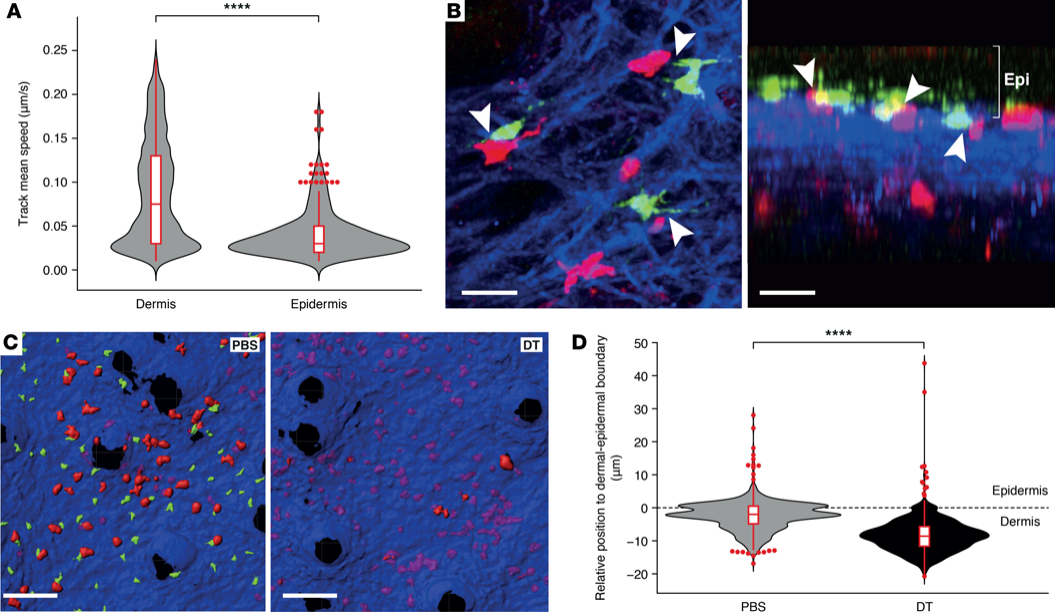
LCs are required for T_E_ migration into the epidermis. (**A**) Track mean speed of MataHari CD8^+^ T cells in dermis and epidermis of male BMT recipients on day 8. Data derived from 3 mice in 3 independent experiments. ****P ≤ 0.0001 by 2-tailed Mann-Whitney test. (**B**) Representative images of donor T cell skin infiltration pattern in early acute GVHD, showing signal overlap for donor-derived MataHari CD8^+^ T cells (red), host-derived LCs (green), and second harmonic signal (blue) (left: maximum *Z*-stack projection; right: *y*-*z* orthogonal view). Scale bars: 20 μm. Donor CD8^+^ T cells accumulated in the epidermis (Epi) where they established close contacts (arrow heads) with host LCs. (**C**) Representative images and (**D**) summary data showing position of MataHari T cells (red) in relation to the epidermis-dermis boundary (blue) in the presence or absence of LCs. Data derived from 4 mice in 2 independent experiments. ****P ≤ 0.0001 by 2-tailed Mann-Whitney test. BMT, bone marrow transplantation; DT, diphtheria toxin; Epi, epidermis; GVHD, graft-versus-host disease; LC, Langerhans cell; T_E_, effector T cell.

Tissues are populated by resident antigen-presenting cells (APCs), the functional specification of which is unique to each tissue site. In the skin, LCs are the only resident myeloid population in the epidermis; following allogeneic BMT, they initially remain of host origin by virtue of their radio-resistance and capacity for local self-renewal (32, 33). We have previously shown that LCs can regulate T cell activation (34); however, a direct role of these APCs in reprogramming T_E_ in situ has not yet been demonstrated. Expression levels of the Notch ligands (specifically Delta-like ligand 4 and Jagged 1) were found to be significantly higher in LCs than other non-LC populations in the epidermis of mice developing GVHD (Supplemental Figure 5A). Furthermore, in vitro Notch blockade abrogated the capacity of male LCs to stimulate IFN-γ generation by activated MataHari T cells (Supplemental Figure 5B). Therefore, we asked whether interaction with resident LCs in the epidermis was responsible for T_E_ reprogramming, specifically for inducing the epidermis-specific M28 gene cluster. Live multiphoton imaging on day 8 demonstrated that trafficking T_E_ sharply reduced their velocity as they moved from the dermis to the epidermis (Figure 6A) and the majority (65%, *n* = 120 cells tracked in 2 independent experiments) formed close contacts with radio-resistant, host-derived LCs in the basal epidermis (Figure 6B). Too few donor-derived LCs were present at this time point to identify any significant interactions with incoming T_E_ (Supplemental Figure 6A). To test the role of LCs in regulating T_E_ accumulation within the epidermis, we induced specific and long-lasting depletion of host-derived LCs with diphtheria toxin (DT) 20 days prior to F→M Langerin.DTR BMT (35, 36). Depletion of host LCs dramatically reduced T_E_ accumulation in the epidermis, with the majority of remaining skin T_E_ now being present within the dermis (Figure 6, C and D).

**Figure 7:**
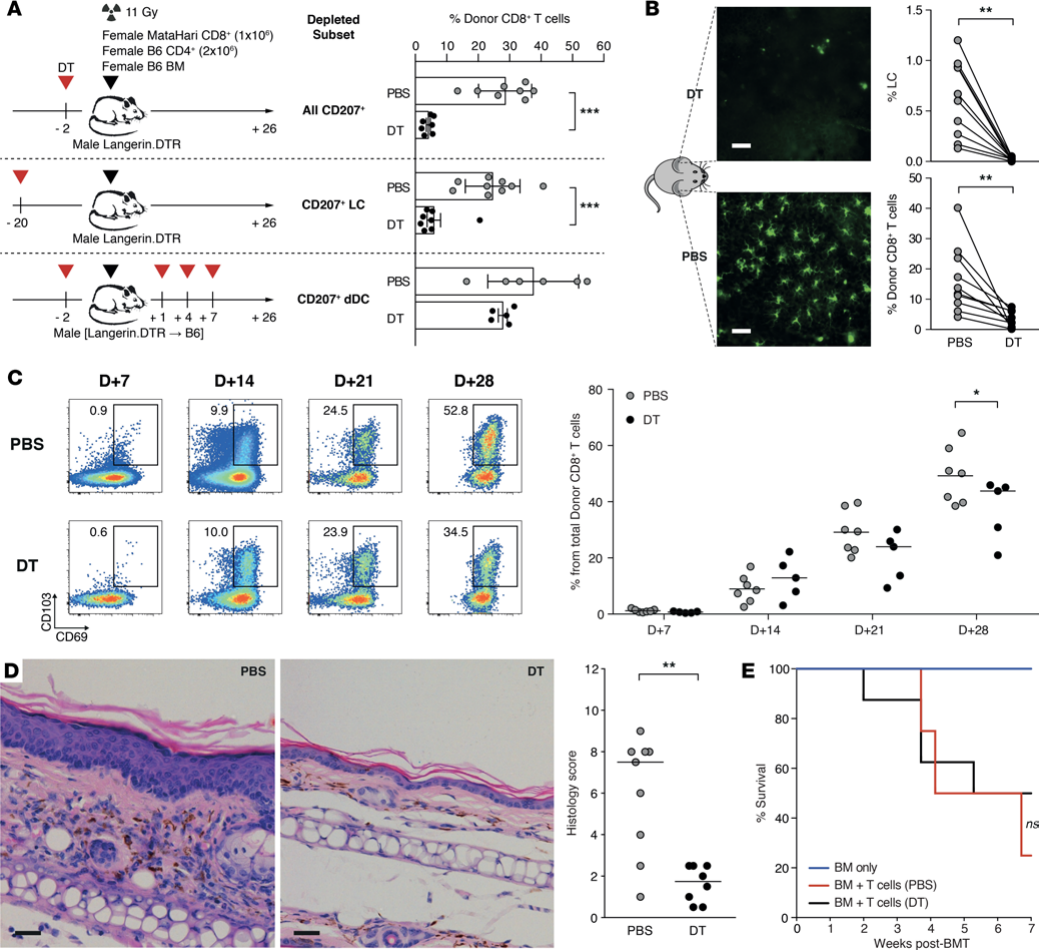
T_E_ pathogenicity in skin is triggered by migration to the epidermis and interaction with LCs in situ. (**A**) Graphs showing mean ± SD epidermal accumulation of MataHari T cells following F→M BMT according to the presence or absence of CD207^+^ cells in the host (*n* = 5–9/group). Timing of DT or PBS treatment determined depletion or otherwise of different subsets of host CD207^+^ populations from the skin (all CD207^+^ *n* = 7–8/group, LC only *n* = 8–9/group, or CD207^+^ dDC only *n* = 5–6/group) in male Langerin.DTR or established (male Langerin.DTR→B6 male) bone marrow chimeras used as BMT recipients. ***P ≤ 0.001 by 2-tailed Mann-Whitney *U* test. (**B**) Left: Representative images of immunofluorescence staining of epidermal sheets showing unilateral LC (green) depletion achieved through intradermal injection of DT (left ear, top image) or PBS (right ear, bottom image). Scale bars: 50 μm. Right: Graphs show summary data for mean ± SD of LC numbers (top) and epidermal T_E_ numbers (bottom) in each ear at day 7 (*n* = 10). **P ≤ 0.01 by 2-tailed Wilcoxon’s matched-pairs signed-rank test. (**C**) Evolution of the T_RM_ phenotype of epidermis-located MataHari T cells in the presence (PBS) or absence (DT) of LCs (left: representative FACS plots of CD69 and CD103 expression over time; right: summary data). *P ≤ 0.05 by 2-tailed Wilcoxon’s rank-sum test. (**D**) Left: Representative images of H&E staining of skin samples from male Langerin.DTR allo-BMT recipients treated with PBS or DT. Right: Summary data of the histopathologic severity score (lines represent median). **P ≤ 0.01 by Mann-Whitney *U* test. (**E**) Survival of F→M BMT recipients according to the presence or absence of LCs (BMT + T cells ± DT, *n* = 8/group) or BMT no–T cell controls (*n* = 3). Log-rank Mantel-Cox test. BM, bone marrow; BMT, BM transplantation; dDC, dermal dendritic cell; DT, diphtheria toxin; D+, number of days after BMT; Epi, epidermis; H&E, hematoxylin and eosin; LC, Langerhans cell; T_E_, effector T cell; T_RM_, resident memory T cell.

To test whether LCs were the only Langerin-expressing population responsible for T_E_ accumulation, we performed additional experiments involving specific depletion of host-type CD103^+^Langerin^+^ DCs (using repeated peritransplant injections of DT to established [Langerin.DTR male→B6 male] BM chimeras undergoing a second transplant) and demonstrated no effect on T_E_ accumulation in the epidermis (Figure 7A). Host LCs were similarly required for epidermal T_E_ accumulation in the B6→129 model and in an adaptation of the F→M model involving transfer of HY-specific CD4^+^ T cells expressing the Marilyn TCR (37), indicating that this requirement was conserved in independent models (Supplemental Figure 6B). Given that LCs are highly motile cells (36), we also tested whether their requirement for T_E_ accumulation occurred because of direct communication in the epidermis or could be explained by interactions taking place elsewhere, for example in the draining LNs. As shown in Figure 7B, unilateral depletion of LCs in the left ear at the time of F→M Langerin.DTR BMT led to reduced T_E_ accumulation in the left but not in the right ear, indicating that the requirement for LCs occurred in situ. The phenotype of epidermis-located MataHari T cells showed increasing divergence over time from their counterparts located in SLOs, with the former differentiating into CD103^+^CD69^+^ resident memory T cell–like (T_RM_-like) cells several weeks following the induction of GVHD (at day 21, 24.9% ± 8.7% in epidermis versus 2.4% ± 1.4% in LNs, *n* = 7/group pooled from 2 experiments, *P* = 0.02, 2-tailed Wilcoxon’s rank-sum test). We therefore considered whether host LCs would influence this process. As shown in Figure 7C, depletion of LCs also led to an eventual reduction in T_RM_ differentiation, as evidenced by the proportion of epidermal MataHari T cells with a dual CD103^+^CD69^+^ phenotype at day 28 after BMT. Depletion of host LCs had a local effect blocking the progression of skin immunopathology (Figure 7D) but had no impact on systemic disease in terms of survival (Figure 7E) or the histological grade of GVHD in other organs (data not shown).

**Figure 8:**
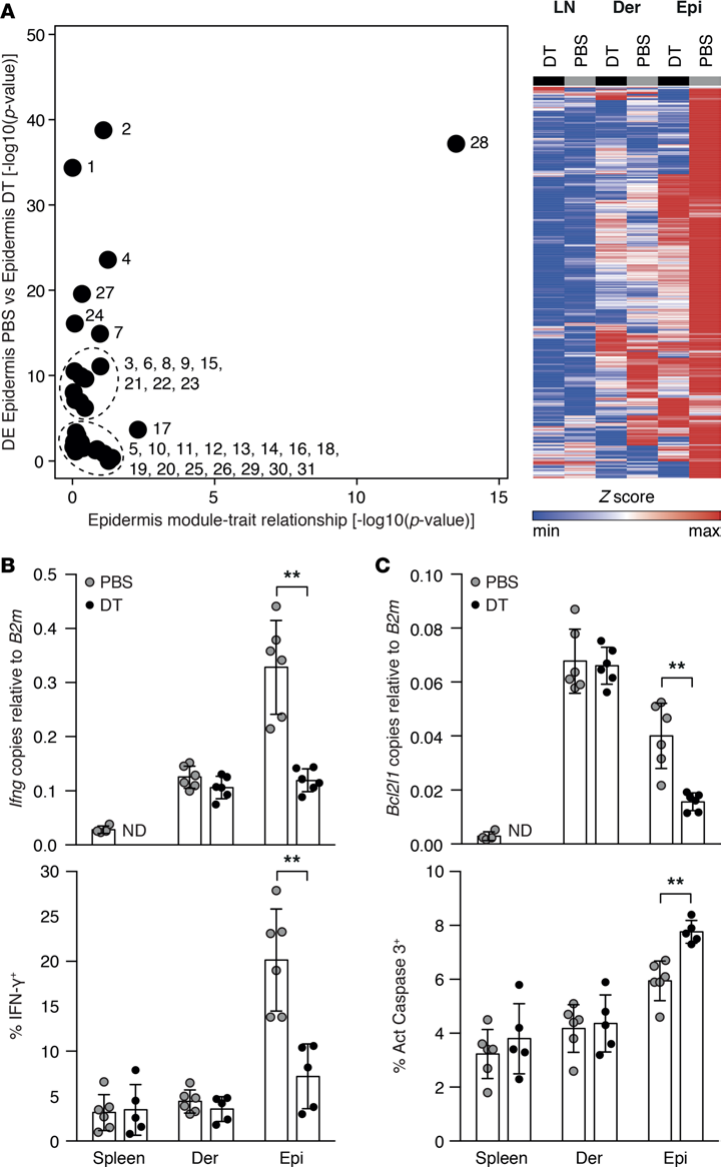
LCs are required to induce the pathogenic gene cluster M28. (**A**) Left: Graph of weighted gene coexpression network analysis-defined modules showing *P* values for correlation to epidermis (*x* axis) and differential expression according to presence or absence of LCs (*y* axis). Right: Heatmap showing the relative expression of M28 genes in the LNs, dermis, and epidermis in the presence (PBS) and absence of LCs (DT). (**B**) Assessment of effector function (left: *Ifng* gene and IFN-γ protein expression) and (**C**) survival/apoptosis (right: *Bcl2l1* expression and caspase 3 activity) of skin-infiltrating donor CD8^+^ T cells in the presence (PBS) and absence of LCs (DT), (*n* = 5–6/group, all graphs showing mean ± SD). **P ≤ 0.01 by Mann-Whitney *U* test. DE, differential expression; Der, Dermis; DT, diphtheria toxin; Epi, epidermis; LC, Langerhans cell; LN, lymph node; ND, not detected.

Given the critical role of LCs in controlling local T_E_ accumulation and tissue injury, we reasoned that gene modules driving the pathogenic process should also be differentially regulated upon LC depletion. To test this hypothesis, we developed a differential expression analysis (modDE) that tests not only whether a module shows an excess of differentially expressed genes, but also whether the differential expression is consistent with the correlation structure identified in the WGCNA analysis (see Supplemental Methods). With this tool, we assessed the differential expression status of all 31 WGCNA-derived modules in purified T_E_ from the epidermis of F→M BMT recipients in the presence or absence of LCs. Consistent with our hypothesis, the M28 gene cluster was unique in being both (a) expressed in the epidermis and (b) showing a high level of differential expression in the presence or absence of LCs (modDE *P* < 10^–30^, Figure 8A). We excluded any possible artifact due to contamination with LCs in nondepleted versus depleted conditions by demonstrating the lack of LC-related gene profiles in the former conditions (Supplemental Methods). We further corroborated these findings by evaluating gene expression (in purified T_E_) or protein expression (by flow cytometry of T_E_ in cell suspensions) of M28 driver genes in the presence or absence of LCs. Thus, *Ifng* mRNA and IFN-γ protein were reduced in epidermal T_E_ in the absence of LCs (Figure 8B). Absence of host LCs also reduced epidermal T_E_ expression of *Bcl2l1*, another M28 driver gene, and this was associated with a local increase in apoptosis as detected by activated caspase 3 staining (Figure 8C). Thus, host LCs are critical for in situ–reprogramming T_E_ as they move from the dermis to the epidermis and for conferring a capacity to induce tissue injury.

## Discussion

Our network analysis shows that peripheral tissue instruction is critical in dictating T_E_ pathogenicity in GVHD. Anatomical divergence of T_E_ programming in GVHD was independent of variation in antigen density at different locations or a skewed TCR repertoire of infiltrating T cells. Furthermore, the concentration of proinflammatory programs in T_E_ from peripheral tissues was not due to the selective enrichment of T cells that had undergone optimal activation in LNs. Instead, the heterogeneity of T_E_ populations according to their position occurred through mechanisms that were primarily tissue autonomous. In vivo perturbation of our experimental BMT system through LC depletion combined with module-based differential expression demonstrated that LCs directly regulated T_E_ pathogenicity in situ within the epidermis. Together, our data show that peripheral tissues create microanatomical niches critical for local injury in GVHD.

To attain an unbiased overview of T_E_ programming according to location, we performed a transcriptional coexpression network analysis. While experimental constraints limited the sample size of the study and consequently its statistical power, this network analysis was sufficiently robust to identify meaningful coexpressed gene modules. Hence, these results provided a unique platform to understand a complex cellular system during the development of immunopathology and to assess its conservation across species. Using our unbiased analytical pipeline, we were able to reveal gene clusters (e.g., M28 expressed by T_E_ in the epidermis) that were central to the development of tissue injury in GVHD and did not correspond to previously identified programs. We observed a high level of conservation for the epidermis-specific gene modules both during a polyclonal murine T cell response against multiple minor histocompatibility antigens and in T cells isolated from human patients with skin GVHD. Of note, despite our observation that target organ T_E_ constituted the most proinflammatory populations, previously defined transcriptional signatures for Tc17 or Tc1 did not relate to cell position in the host. This discrepancy probably reflects the fact that the published transcriptional profiles are derived primarily from T_E_ isolated from lymphoid tissues, thus highlighting the current information gap for tissue-related programs. These findings therefore challenge whether effector programs defined by evaluation of T_E_ isolated from blood or lymphoid organs can be relied on to explain mechanisms leading to organ immunopathology; it is likely that such analyses will miss definitive groups of coregulated genes that fully equip T cells to induce injury in peripheral tissues.

In the skin, we found that LCs instructed local T cell pathogenicity in 3 independent models of GVHD following MHC-matched, minor antigen–mismatched transplantation (in both CD8^+^ and CD4^+^ T cell–dependent models of GVHD following F→M BMT, and following B6→129 BMT), thus overriding their steady-state role in promoting tolerance (38). A similar role for LCs has been reported following MHC-mismatched BMT (33, 34). Our findings are also consistent with a previous study showing the redundancy of host LCs in promoting systemic GVHD (39) but are at variance with a single report using human Langerin.DTA BMT recipients that lack LCs through their lifespan and where skin GVHD was unaffected (40). It is possible that the requirement for LCs may depend on the extent to which antigens targeted in GVHD are concentrated in the skin; alternatively, there may be as-yet-undefined changes to baseline immunity in the long-term absence of LCs (41). We could also further assign pathogenicity to a specific T_E_ gene cluster induced by LCs by evaluating differential module expression in an LC depletion experiment. The integration of this follow-up analysis with the WGCNA data required the development of modDE; its potentially novel feature is the ability to combine gene-based *P* values with the direction of effect for each gene, hence ensuring that significant modules are the ones for which the pattern of up- or downregulation is consistent with the correlations structure identified by WGCNA. Through their functions as sensors capable of integrating complex environmental cues, resident or recruited APCs are well placed to act as a conduit for reprogramming T_E_ in nonlymphoid tissues. In the context of infection, cognate interactions with monocyte-derived CD11c^+^ DCs recruited to inflamed tissues are necessary for amplification of local cytokine generation and proliferation by T_E_ (42, 43) or T_RM_ cells (44). An APC-dependent checkpoint may therefore be important for fine tuning of the T_E_ response, according to the type of infection and the levels of antigen present locally (45). Our finding that this process can become corrupted during the development of GVHD is supported by the involvement of other CD11c^+^ APC populations recruited to the sites of T cell–mediated immunopathology in autoimmune (46–48) or infection-related (49) inflammation. Collectively, these data support a model of sequential differentiation in which T cell activation in SLOs leads to proliferation and upregulation of tissue-specific homing receptors, but full effector competence requires a second hit from an APC following T_E_ entry to inflamed tissues.

Although host LCs were identified as critical initiators of tissue injury in skin GVHD, other instructional mechanisms will operate at other sites. In the context of the inflammatory response generated during the initiation of GVHD, it is possible that nonhematopoietic cells can substitute for APCs (39, 50), although this may be dependent on the type of immune response or tissue involved. Independent of interaction with APCs, inflammatory cytokines (e.g., IL-12 and type 1 interferons) may also act *in trans* to directly influence proximal TCR signaling and antigen sensitivity of responding T cells (51). In the GVHD models we employed in this study, irradiation-induced injury to epithelial barriers drives profound activation of the innate immune system through recognition of damage-associated or pathogen-associated molecular patterns (52). It is therefore likely that the repertoire of immune cells within individual nonlymphoid organs and interaction with the local microbiota will additionally shape the cytokine microenvironment and hence, the differentiation patterns of T_E_ (53, 54). Such factors are likely to be relevant to the distinct effector identity observed in T_E_ derived from different target organs.

A non–mutually exclusive mechanism underlying the divergence of T_E_ functions between lymphoid and nonlymphoid organs in GVHD may involve the uneven distribution of counterregulatory pathways present at the initiation of or during propagation of the response (e.g., levels of coinhibition or the presence of regulatory T cells) (12). However, we have observed that many of the pathways upregulated in T_E_ of lymphoid organs are linked to proliferative and metabolic fitness, making it unlikely that global suppression of the T cell response in lymphoid organs can easily account for the differences with peripheral tissues. In fact, coinhibitory pathways are generally induced in peripheral tissues in response to inflammation under conditions where they are evidently insufficient to completely curtail a local effector response (55–57). Thus, other mechanisms independent of counterregulation are likely to explain the tissue-specific reprogramming of pathogenic T_E_. Indeed, the M28 gene cluster activated in T_E_ by LCs required Notch signaling and late interruption of this pathway prevented local T_E_ function and differentiation into T_RM_-like cells. This finding of a role for Notch in dictating the differentiation in peripheral tissues has strong parallels to the recent demonstration that Notch is also required to maintain functional T_RM_ cells within the lung epithelia (58) or to drive local T cell pathogenicity in large-vessel vasculitis (59). Thus, although early Notch signaling is required in the first 48 hours following BMT for optimal priming of CD8^+^ T_E_ in GVHD (29, 60), it is likely that cells recruited to tissues will later encounter a distinct repertoire of Notch ligands that can direct further changes in state or fate. Such encounters may also be critical to driving treatment resistance to immunosuppressive drugs, as suggested by our finding of GC-resistance signatures in T_E_ from patients at the onset of acute skin GVHD.

In conclusion, we have shown that the location of T_E_ in the host is the critical driver of pathogenicity in GVHD. In the epidermis, LCs are critical for the reprogramming of incoming T_E_ and the triggering of local injury. The concordance of our experimental murine and human data in the skin support the notion that a tissue-based dissection of T cell immunity will better promote design of precision therapies for GVHD that block immunopathology while avoiding global immune suppression.

## Methods

### Mice.

C57BL/6 and 129/Sv mice were purchased from Charles River Laboratories and bred in house by UCL Biological Services. C57BL/6 Langerin.DTREGFP (Langerin-DTR) mice (36) were provided by Bernard Malissen and Adrien Kissenpfennig (Université de la Méditerrannée, Marseille, France) and bred in house. C57BL/6 TCR-transgenic anti-HY MataHari mice (61) and Marilyn mice (37) were provided by Jian Chai (Imperial College London, London, UK) and bred in house. hLangerin-Cre-YFP mice (62) were provided by Daniel Kaplan (University of Pittsburgh) and bred in house. 129.Langerin.DTR mice were bred in house by coupling 129/Sv and C57BL/6 Langerin.DTREGFP mice. MataHari-CD2-DsRed mice were bred in house by coupling C57BL/6 MataHari and C57BL/6 CD2-DsRed (63) provided by Mark Coles (University of York, York, UK). Animals used as recipients for BMT were 10–20 weeks old, and donors were 8–16 weeks old.

### BMT.

BMT was performed as described previously with minor modifications (20). Briefly, recipient mice were lethally irradiated (11 Gy total body irradiation, split into 2 fractions over a period of 48 hours, at day –2 [D–2] and day 0 [D0]) and reconstituted 4 hours later with 5 × 10^6^ BM cells, 2 × 10^6^ CD4^+^ splenocytes, and 1 × 10^6^ CD8^+^ splenocytes, administered by intravenous injection through the tail vein. Isolation of CD4^+^ and CD8^+^ T cells was performed by immunomagnetic selection of CD4^+^ or CD8^+^ splenocytes using Manual MACS Cell Separation Technology (QuadroMACS Separator, LS columns, CD4 [L3T4] MicroBeads, CD8a [Ly-2] MicroBeads; Miltenyi Biotec), according to the manufacturer’s instructions. B6→129 model: 129/Sv or 129.Langerin.DTR male mice (CD45.2^+^/Thy-1.2^+^) were used as recipients and C57BL/6 female mice (CD45.1^+^/Thy-1.1^+^) were used as BM and splenocyte donors (allo-BMT group); or C57BL/6 female mice (CD45.2^+^/Thy-1.2^+^) were used as recipients and C57BL/6 female mice (CD45.1^+^/Thy-1.1^+^) were used as BM and splenocyte donors (syn-BMT group). F→M model: C57BL/6 male (CD45.2^+^/Thy1.2^+^), Langerin-DTR, or hLangerin-Cre-YFP male mice were used as recipients, C57BL/6 female mice (CD45.2^+^/Thy1.2^+^) were used as BM and CD4^+^ splenocyte donors, and MataHari or MataHari-CD2-DsRed female mice (CD45.2^+^/Thy1.1^+^) were used as CD8^+^ splenocyte donors (allo-BMT); C57BL/6 female mice (CD45.2^+^/Thy1.2^+^) were used as recipients, C57BL/6 female mice (CD45.2^+^/Thy1.2^+^) were used as BM and CD4^+^ splenocyte donors, and MataHari female mice (CD45.2^+^/Thy1.1^+^) were used as CD8^+^ splenocyte donors (syn-BMT).

### LN lymphocyte egress blocking experiments.

Blocking the egress of activated lymphocytes from the LNs to the peripheral tissues was achieved through treatment of transplanted animals with the sphingosine-1-phosphate antagonist, fingolimod (FTY720; Sigma-Aldrich), as previously described (64). Briefly, BMT recipients were injected intraperitoneally with 1.0 mg/kg FTY720 daily, from D+3 to D+7 (experiment terminus); control subjects received an equivalent volume of saline.

### LC depletion.

Selective depletion of CD207^+^ cells was achieved through treatment of Langerin‑DTR mice or (male Langerin-DTR→male B6) BM chimeras with DT (Sigma-Aldrich) at different time points. In Langerin-DTR recipients, a single injection at D–2 was used for depletion of all CD207^+^ cell populations, whereas a single injection at D–20 was used for specific depletion of LCs. In (male Langerin‑DTR→male B6) recipients, DT injections were given at D–2, D+1, D+4, and D+7 for specific depletion of CD207^+^ dermal DCs (dDCs). For systemic depletion, 400 ng DT was administered intraperitoneally; for localized LC depletion, animals received intradermal injections of 25 ng of DT in dorsal and ventral sides of the left ear (total DT dose, 50 ng). Control subjects received an equivalent volume of saline.

### Interruption of Notch signaling.

In vitro assays: LCs were differentiated from BM as described previously (65) with some modifications. Briefly, femurs and tibias from C57BL/6 male and female mice were flushed, and recovered cells were counted, resuspended at 10^6^ cells/ml in complete T cell medium (RPMI, 1% FCS, 1% L-glutamine, 1% penicillin-streptomycin, and 1% HEPES; Lonza) with 20 ng/ml recombinant murine GM-CSF (PeproTech) and 5 ng/ml recombinant murine TGF-β1 (eBioscience), cultured at 37°C for 3 days, and FACS isolated (CD11c^+^ MHC class II^+^ EpCAM^+^). MataHari CD8^+^ T cells were activated overnight in complete T cell medium with 2 μg/ml concanavalin A and 1 ng/ml IL-7, and plated in a 1:1 ratio with either female or male LCs. Cells were treated with 10 μM LY411575 (Sigma-Aldrich) or vehicle (complete T cell medium) and cocultured for 18 hours at 37°C. In vivo experiments: Interruption of Notch signaling was achieved through treatment of transplanted animals with 5 mg/kg/day LY411575 given intraperitoneally, from D+5 to D+7 (experiment terminus); control subjects received an equivalent volume of saline.

### Tissue and organ harvest.

Blood samples were collected into heparinized tubes by venipuncture of the lateral tail vein, for interim analysis, or by cardiac puncture under terminal anesthesia, at the experiment terminus. Following intracardiac perfusion with 20 ml of cold PBS to remove the blood from the vasculature, the organs of interest were harvested and stored in harvest medium (PBS, 2% FCS, and 1% penicillin-streptomycin; Lonza) on ice. Processing of the samples was started within 2 hours from collection. Harvested organs included spleen, peripheral LNs (cervical, axillary, brachial and inguinal), mesenteric LNs, tibias and femurs, liver, small intestine (from 0.5 cm below the stomach to 1 cm above the cecum), and skin (body and ears).

### Histological evaluation.

Histological evaluation of GVHD in the skin, gut, and liver was performed single blinded following the scoring system previously described (66).

### Statistics.

Statistical analysis was performed using GraphPad Prism version 6.00 for Mac OsX. Significance was assessed using a 2-tailed Mann–Whitney *U* test or 2-tailed Wilcoxon’s signed rank-sum test for paired comparisons. For multiple comparisons, a 1-way ANOVA with Holm-Sidak post hoc test was used. Survival curve comparison was performed using the log-rank Mantel-Cox test. A *P* value ≤ 0.05 was taken to indicate a significant difference between groups; only statistically significant differences are marked in the figures. Sample sizes (number of animals), *n*, definition of center, dispersion and precision measures are indicated in the figure legends. Microarray and RNAseq data analysis is described in Supplemental Methods.

### Study approvals.

All procedures were conducted in accordance with the UK Home Office Animals (Scientific Procedure) Act of 1986, and were approved by the Ethics and Welfare Committee of the Comparative Biology Unit, Hampstead Campus, UCL, London, UK.

Human studies of patients developing acute pattern GVHD following allogeneic hematopoietic stem cell transplantation were approved following ethical review by the NHS Health Research Authority (REC reference: 14/NE/1136; IRAS project ID: 129780) and all patients provided written informed consent.

### Data availability.

The data generated in this paper have been deposited in ArrayExpress; accession numbers are E-MTAB-5378, E-MTAB-5379, E-MTAB-5380, and E-MTAB-5381.

## Author contributions

PS, MC, VP, CLB, and RC contributed to the study concept and design, development of methodology, analysis and interpretation of data, and writing and review of the manuscript. PS, SC, SB, TC, LJ, CT, IRF, HW, TKM, and SH contributed to the development of methodology and acquisition/interpretation of data. SW, CL, and SD acquired data. DHK provided technical and material support. VP, CLB, and RC contributed equally to preparation of the manuscript.

## Supplementary Material

Supplemental data

Supplemental Table 1

Supplemental Table 2

Supplemental Table 3

Supplemental Table 4

## Figures and Tables

**Table 1:**
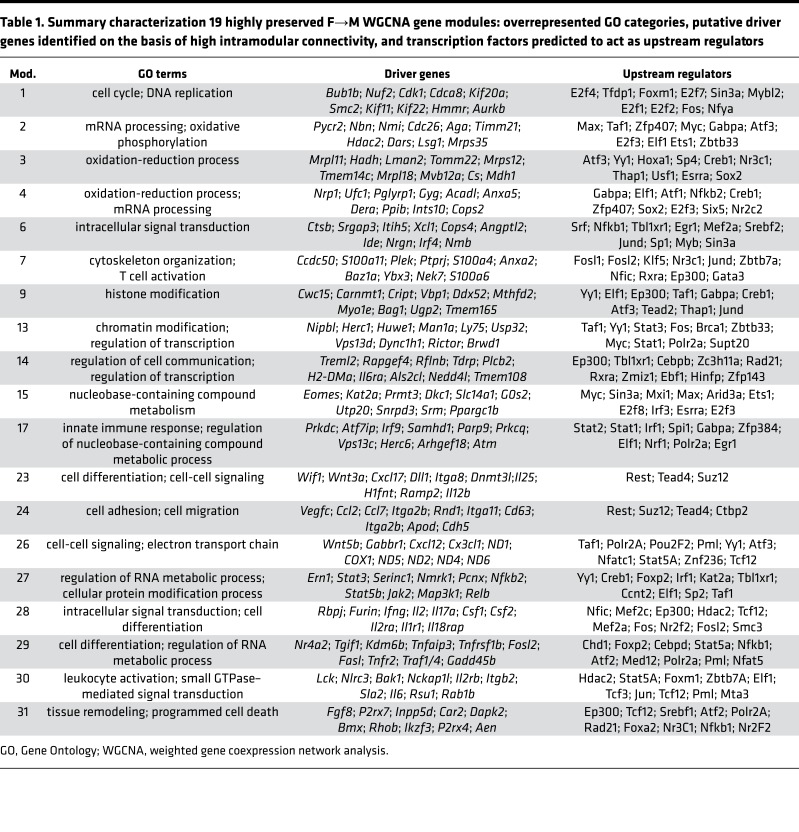
Summary characterization 19 highly preserved F→M WGCNA gene modules: overrepresented GO categories, putative driver genes identified on the basis of high intramodular connectivity, and transcription factors predicted to act as upstream regulators

## Version 1 03/08/2018

Electronic publication
