# Supplementary material for: Peripheral tissues reprogram CD8+ T cells for pathogenicity during graft-versus-host disease
Source: JCI Insight. 2018 Mar 8;3(5):e97011. doi: 10.1172/jci.insight.97011 (PMC5922296; doi:10.1172/jci.insight.97011)
Supplement: Supplemental data [file jciinsight-3-97011-s001.pdf]

ONLINE SUPPLEMENTAL MATERIAL

Supplemental Methods include information regarding isolation of murine immune cells, flow cytometry analysis and cell sorting, sample preparation of gene expression analysis, multi-photon *in vivo* imaging, immunofluorescence staining of epidermal sheets, microarray and RNAseq analysis, data set pre-processing and gene subtraction, sample relationship visualization, differential gene expression, GSEA, ssGSEA, WGCNA, prediction of upstream master regulators, overrepresentation analysis, and module-based differential expression analysis. *Supplemental Table 1*, included in a separate Excel file, lists for the 19 highly preserved F→M WGCNA gene modules, and the 2 skin-specific B6→129 WGCNA modules: the over-represented Gene Ontology categories (Supplemental Table 1A), the putative driver genes identified on the basis of high intra-modular connectivity (Supplemental Table 1B), and the transcription factors predicted to act as upstream regulators (Supplemental Table 1C). *Supplemental Table 2*, included in a separate Excel file, lists the clinical details for the 5 HSCT patients included in this study. *Supplemental Table 3*, included in a separate Excel file, contains the results from the pre-processing performed to test for LC contaminants in LC-replete versus LC-depleted epidermal samples (Supplemental Table 3A), and lists the intestine- and skin-specific genes subtracted from the analysis (Supplemental Table 3B). *Supplemental Figure 1* shows the visualization of sample relationship without gene subtraction. *Supplemental Figure 2* shows the overlap between T_E gene expression profiles in the F→M and B6→129 experimental models, and the similarities between F→M and B6→129 based WGCNA modules. *Supplemental Figure 3* depicts the analytical pipeline followed, based upon correlation network analysis and downstream validation. *Supplemental Figure 4* shows the network representation of the B6→129 skin-specific modules MD and ME, and their overlap with the F→M skin-specific module M28. *Supplemental Figure 5* shows the expression of JAG1 and DLL4 Notch ligands by LC compared to non-LC populations in the epidermis of mice developing GVHD, and the effect of *in vitro* Notch blockade on the capacity of male LC to stimulate IFN-γ generation by activated MataHari T cells. *Supplemental Figure 6* shows the kinetics of LC host-to-donor turn over and the effect host LC depletion upon T_E accumulation in the epidermis in independent murine BMT models of GVHD.

Supplemental Methods

Isolation of murine immune cells

a) Lymph nodes and spleens – To prepare cell suspensions from spleens and lymph nodes, the freshly removed organs were mashed and passed through a 40 µm cell strainer; red blood cells were removed by isotonic lysis with ammonium chloride (ACK Lysing Buffer; Lonza, UK). Cells were re-suspended in FACS buffer (PBS, 2% FCS, 2 mM EDTA; Lonza UK) for counting and immunolabelling.

b) Bone marrow – To isolate bone marrow cells, both epiphyses of the long bones of the hind limbs were cut and the bone marrow was flushed out with FACS buffer. The cell suspension was filtered through a 40 µm cell strainer and red blood cells were removed by isotonic lysis with ammonium chloride. Cells were re-suspended in FACS buffer for counting and immunolabelling.

c) Blood – Erythrocytes were removed from whole blood samples by hypotonic lysis with distilled water. Cells were re-suspended in FACS buffer for counting and immunolabelling.

d) Small intestine – The entire small intestine was flushed and rinsed with 40 ml of ice cold harvest medium (PBS, 2% FCS, 1% penicillin-streptomycin; Lonza, UK), and sectioned into ~0.5 cm pieces. The intestinal pieces were incubated with detaching medium (HBSS, 5% FCS, 1% penicillin-streptomycin, 5 mM EDTA; Lonza, UK) at 37°C with shaking at 150 rpm for 60 min. The supernatant, containing the IEL, was passed sequentially through a 100 µm and a 40 µm cell strainer and enriched for lymphocytes by density gradient centrifugation with Ficoll Paque™ Plus (GE Healthcare, UK). The intestinal pieces were further incubated in digestion medium (RPMI, 2% FCS; Lonza, UK; 200 U/ml collagenase IV; LifeTechnologies, USA; 200 U/ml DNAse I; Sigma, UK) at 37°C with shaking at 150 rpm for 60 min, dissociated and passed sequentially through a 100 µm and a 40 µm cell strainer. The cell suspension, containing the LP cells was enriched for lymphocytes by density gradient centrifugation with Ficoll Paque™ Plus. Cells were re-suspended in FACS buffer for counting and immunolabelling.

e) Skin – Epidermal and dermal immune cells were isolated from the skin in accordance with the protocol described by Henri *et al.* (1), with minor modifications. Briefly, the body skin was cut into ~1x1 cm pieces, after having been shaved and the subcutaneous fat removed, and the ears were

split in two parts (ventral and dorsal). The pieces of skin were incubated overnight in dispase medium (HBSS, 2% FCS; Lonza, UK; 2.5 mg/dl dispase II; Sigma, UK) at 4°C, and the epidermal and dermal sheets were separated and cut into ~0.5 cm fragments. The epidermal fragments were vortexed, mashed and passed sequentially through a 100 µm and a 40 µm cell strainer to disintegrate the remaining tissue and create a cell suspension. The dermal fragments further incubated in digestion medium at 37°C with shaking at 150 rpm for 60 min, dissociated and passed sequentially through a 100 µm and a 40 µm cell strainer. Cells were re-suspended in FACS buffer for counting and immunolabelling.

Flow cytometry

Cells were plated out at up to $1x10^6$ cells per well in a 96 well conical bottom plate and incubated with 2.4G2 antibody at 4°C for at least 10 min to block Fc receptors. For cell surface immunolabeling, cells were incubated with the fluorochrome-conjugated antibodies diluted in 100 µl of FACS buffer at 4°C for 20 min in the dark – CD4 (GK1.5, eBioscience, USA), CD8a (53-6.7, BD Biosciences, Germany), CD11b (M1/70, eBioscience, USA), CD45 (30-F11, BioLegend, USA), CD45.1 (A20, BD Biosciences, Germany), CD45.2 (104, eBioscience, USA), Thy-1.1 (HIS51, eBioscience, USA), Thy-1.2 (53-2.1, BD Biosciences, Germany), EpCAM (G8.8, eBioscience, USA), MHC class II I-A/I-E (M5/114.15.2, eBioscience, USA), DLL4 (HMD4-1, BioLegend, USA), JAG1 (HMJ1-29, BioLegend, USA), LPAM-1 (DATK32, BioLegend, USA), Vβ8.3 TCR (1B3.3, BD Biosciences, Germany). When intracellular staining was required, after having performed cell surface immunolabelling, samples were washed twice with FACS buffer, fixed in 100 µl of fixation solution (BD Cytofix/Cytoperm solution; BD Biosciences, UK) for 15 min at 4°C in the dark, washed twice with permeabilization solution (BD Perm/Wash™ buffer; BD Biosciences, UK), and incubated with the fluorochrome-conjugated antibodies diluted in 100 µl of permeabilization solution at 4°C for 30 min in the dark – Active Caspase-3 (C92-605, BD Biosciences, Germany), CD207 (eBioL31, eBioscience, USA), IFN-γ (XMG1.2, BD Biosciences, Germany). For detection of cytokine production, cells were treated with brefeldin A (Sigma, UK) for 2 h at 37°C, prior to immunolabelling. Samples were washed twice with FACS buffer and re-suspended in 300 µl of FACS buffer for immediate analysis by flow cytometry. For

dead cell exclusion, 2 µl of propidium iodide (LifeTechnologies, USA) was added to the unfixed samples prior to analysis. For assessment of cell proliferation, cells were stained with carboxyfluorescein succinimidyl ester (CellTrace™ CFSE Cell Proliferation Kit, Invitrogen, USA), according to the manufacturer's instructions. Multicolor flow cytometry data acquisition was done with BD LSRFortessa and BD LSR II cell analyzers equipped with BD FACSDiva v6.2 software (BD Biosciences, Germany). Fluorescence activated cell sorting was performed on a BD FACSAria equipped with BD FACSDiva v5.0.3 software (BD Biosciences, Germany). All samples were maintained at 4°C for the duration of the sort. Sort purity was accessed for all samples and only those with purity ≥ 95% were used for RNA extraction. Cells were sorted directly into Buffer RLT (QIAGEN, USA) with 1% 2-β-mercaptoethanol (Sigma, UK), disrupted through vortexing at 3200 rpm for 1min, and immediately stored at -80°C until further processing. Three biological replicates were obtained for every tissue from 3 independent experiments, each containing a minimum of 4000 cells (pooling where necessary from multiple mice from individual experiments). Flow cytometry data were analyzed with FlowJo X v10 (LLC, USA).

Sample preparation for gene expression analysis

RNA was extracted using the RNeasy Micro Kit (QIAGEN, USA) following the manufacturer's protocol. RNA yield, quality and integrity were evaluated using the RNA 6000 Pico kit on an Agilent 2100 Bioanalyser (Agilent Technologies, USA). Only samples with a RNA Integrity Number (RIN) above 8.0 were included in the study. For microarray studies, the Ovation Pico WTA System V2 kit (NuGEN, USA) was used to prepare amplified cDNA from total RNA. Spectrophotometric absorbance of the cDNA products at 260, 280 and 320 nm was determined to assess purity; all samples had an adjusted (A260 – A320):(A280 – A320) ratio > 1.8. Fragmentation and labeling of the cDNA samples was performed using the Encore Biotin Module kit (NuGEN, USA), according to kit instructions, and then hybridized onto GeneChip Mouse Gene 2.0 ST arrays (Affymetrix, USA). Hybridisation was performed in a single batch per experimental system. For RNA-seq studies, RNA samples were amplified using the SMART Seq® v4 Ultra® Low Input RNA Kit. Paired-end sequencing libraries were prepared from the amplified cDNA according to the Nextera® XT DNA library prep protocol, and sequenced (38 base-paired reads)

using an Illumina NextSeq 500 (Illumina, USA). Microarray and RNA-seq studies were performed in collaboration with UCL Genomics.

Multi-photon in vivo imaging

Mice were anesthetized with fentanyl-fluanisone (Hypnorm®; VetaPharma, UK) 0.4 ml/kg and midazolam 5 mg/kg injected IP. Anesthesia was prolonged by injecting additional doses of Hypnorm® (0.3 ml/kg) every 40-60 min. Ears were depilated using NairTM Hair Remover (Church & Dwight, UK). Mice were then placed on a custom-made imaging platform and the ears fixed with a double-sided tape. PBS was placed on top of the ear and a water reservoir, created with a metal ring sealed by a coverslip, was glued on top. Mice were then placed in the thermostated imaging chamber and movies of 22-60 min were acquired. Images were acquired with the Leica DM6000-CFS multiphoton microscope (Leica Microsystems, UK) enclosed in a dark chamber heated at 37°C with heated air, and the HC FLUOTAR 25x/0.95 N.A. water immersion objective (Leica Microsystems, UK). EYFP and DsRed were excited at 950 nm. The emitted fluorescence was acquired using a hybrid detector (HyD; Leica microsystems, UK). The second harmonic generation, produced by the collagen present in the dermis, was visualized by setting the first HyD channel to a 450-500 nm filter window; the EYFP fluorescence by setting the second HyD channel to 510-565 nm and the DsRed fluorescence by setting the third HyD channel to 575-660 nm. Raw image data were processed with Fiji (ImageJ): drift was corrected with the Fiji plugin "Correct 3D drift" using the SHG channel as reference; a median filter (0.3 pixel) was applied to reduce background noise and autofluorescence was removed using the command Image Calculator to subtract crossover signal between channels:

corrected channel 1 = channel 1 – channel 2 – channel 3

corrected channel 2 = channel 2 – channel 1 – channel 3

corrected channel 3 = channel 3 – channel 1 – channel 2

Compilation images (*z*-stack projection and *yz* orthogonal view) were created with Imaris 8.3 software (Bitplane).

Immunofluorescence staining of epidermal sheets

Epidermal sheets were fixed in ice-cold acetone, blocked in a 0.25% fish gelatin, 10% goat serum solution and stained for LC with CD207 (eBioL31) (eBioscience, USA). Images were acquired with the Leica DMI4000b fluorescence microscope (Leica Microsystems, UK), using the Leica Application Suite Advanced Fluorescence v3.2 software (Leica Microsystems, UK). Raw image data were processed with Fiji (ImageJ).

Microarray analysis

A total of 36 RNA samples from the B6→129 model and 61 RNA samples from the F→M model were used for microarray analysis, representing 3 independent biological replicates for each of the tissues/compartments, treatment conditions and time points. Hybridized arrays were scanned with a GeneChip 3000 7G scanner (Affymetrix, USA) (1 batch for B6→129 samples; 3 batches for F→M samples) and the image data processed to generate .cel files. Expression Console Software, version 1.4.1 (Affymetrix, USA) was used to generate quality control statistics for each sample; samples with a high mean absolute deviation of the residual for a chip versus all chips in the data set (≥ data set average + 2 SD), a high mean absolute relative log expression (≥ data set average + 2 SD) and a low area under the curve (AUC) for a receiver operator curve (ROC) comparing the intron controls to the exon controls (≤ 0.8) were considered to have poor hybridisation quality and excluded from the analysis (1 sample in the B6→129 data set; 3 samples in de F→M data set). Raw sample expression signals were background subtracted, quantile normalized, and the probe level data were summarized using the Robust Multi-array Average algorithm (2, 3) implemented in the oligo BioConductor R package (4). The ComBat algorithm from the *sva* BioConductor R package (5) was employed to adjust for batch effects. Transcripts identified through multiple probes were collapsed based on maximum expression values using the CollapseDataset module of GenePattern software (Broad Institute) (6).

Data set pre-processing and gene subtraction

Pre-processing of the data sets was performed to test for LC contamination in the microarray samples used in experiments shown in Fig. 8. We found no significant difference between LC-

replete and LC-depleted epidermal samples in terms of the levels of expression of LC signature genes (7) (Supplemental Table 3A). To exclude any effect of tissue-specific transcripts derived from non-T cell contamination of individual samples, transcripts identified as highly specific for the intestine and skin in the PaGenBase database [specificity measure (PMS) > 0.9] (8) were subtracted from the entire dataset prior to analysis (Supplemental Table 3B).

RNAseq analysis

FASTQ Toolkit, version 1.0.0 (9), was used for adapter trimming of the reads. Alignment and mapping of all libraries were performed using TopHat Alignment, version 1.0.0 and Cufflinks Assembly & DE, version 2.0.0, selecting *Homo sapiens* hg38 RefSeq gene annotations. Gene expression levels were calculated using the Cufflinks Assembly & DE, version 2.0.0, employing a geometric library normalization method and a fragment bias correction algorithm.

Samples relationship visualization

Multivariate statistical analysis methods implemented in the *stats* R package, in particular multidimensional scaling, were applied to perform dimensionality reduction of the datasets and visualization of the samples relationships. Similarities between groups were evaluated by hierarchical clustering, using average-linkage upon the Pearson's correlation-based dissimilarity matrix; the validity and stability of the clusters was assessed using a non-parametric bootstrap as implemented in the *pvclust* R package (10). Additional hierarchical clustering and heat maps were produced using the matrix visualization and analysis platform GENE-E (Broad Institute, USA).

Differential gene expression

The *limma* BioConductor R package was used to perform analyses of gene differential expression, using an empirical Bayes moderated t-statistic corrected for multiple hypothesis testing using Benjamini-Hochberg procedure, with a cut-off of false discovery rate (FDR) ≤ 0.05, and an absolute fold-change cut-off ≥ 2.0.

Gene set enrichment analysis (GSEA)

Gene Set Enrichment Analysis was performed using the GSEA software with the gene sets derived from the Biological Process Ontology database (C5) defined by the Gene Ontology Consortium, collected in the Molecular Signatures Database (MSigDB v5.1), and the gene sets derived from the modules identified by WGCNA in the F→M model dataset.

Single-sample gene set enrichment analysis (ssGSEA)

ssGSEA was performed using the *GSVA* R package employing the "ssgsea" method (11), as described by Hänzelmann et al. (12). The Tc1 gene signature was derived from Best et al. data set (13) as the top 100 over-expressed genes (fold change ≥ 1.5 and FDR ≤ 0.05) in effector OT-I cells on day 6 post Lm-OVA or VSV-OVA infection in comparison to naïve OT-I cells; the Tc17 gene signature was derived from Gartlan et al. (14) data set as the differentially expressed genes (fold change ≥ 1.5 and FDR ≤ 0.05) between $CD8^{+}YFP^{+}$ and $CD8^{+}YFP^{neg}$ T-cells 7 days after allogeneic transplant; the MDR1+ Th1/Th17 gene signature corresponds to the *ex vivo* transcriptional signature of MDR1+ Th17.1 cells isolated from involved Crohn's disease patient tissue published by Ramesh et al. (15) (Supplemental Table 4).

Weighted gene co-expression network analysis (WGCNA)

Scale-free network topology analysis of microarray expression data was performed using the *WGCNA* R package, as previously described (16, 17). A signed hybrid weighted correlation network was constructed using a Pearson correlation matrix created from the pairwise comparison between all pairs of genes, and a soft thresholding power $\beta=8$. The topological overlap was calculated as a measure of network interconnectedness, and genes were grouped by average linkage hierarchical clustering on the basis of the topological overlap dissimilarity (1-topological overlap). Module eigengenes were calculated using a dynamic tree-cutting algorithm and merging threshold function at 0.25. The modules identified were correlated to the sample traits using a binary vector representation of the tissues of origin and study groups. To validate the microarray analysis, preservation of the WGCNA modules identified in the F→M model dataset was tested against the dataset of the B6→129 model, using the R function

"modulePreservation" in the WGCNA R package, as previously described (18). Results were interpreted according to the following thresholds for $Z_{summary}$: if $Z_{summary} \geq 10$, module "strongly preserved"; if $Z_{summary} \geq 2$ and <10, module "weak to moderately preserved"; if $Z_{summary} < 2$, module "not preserved". Significance of the $Z_{summary}$ scores was calculated by gene permutation testing. Intramodular hub genes, which are genes that have the highest number of connections within a module, were identified on the basis of having eigengene-based connectivity (kME) > 0.8 and gene significance (GS) > 0.2. Visualisation of the modules network of gene connections was accomplished with the Cytoscape v3.5 software (19).

Prediction of upstream master regulators

The Cytoscape plugin iRegulon was used to predict the transcriptional regulatory network underlying each of the modules of co-expressed genes, as described by Janky et al. (20). Briefly, for each gene set, the regulatory sequences in 20 Kb around the transcription start site were analyzed, and motif prediction was performed using an enrichment score threshold of 2.0, a ROC threshold for AUC calculation of 0.03%, and a rank threshold for visualization of 5000. Candidate transcription factors were predicted with a maximal FDR for motif similarity of 0.05.

Overrepresentation analysis

The Web-based Gene Set Analysis Toolkit (WebGestalt), a suite of tools for functional enrichment analysis, was used to identify overrepresented KEGG pathways categories and translate gene lists into functional profiles. Statistical significance of KEGG pathways enrichment was calculated based on hypergeometric distribution statistics, adjusting the false discovery rate using the Benjamini-Hochberg procedure.

Module-based differential expression analysis (modDE)

In order to quantify potentially significant associations at the level of WGCNA-derived gene modules between tissue types and/or between conditions, we developed a gene based association test that takes advantage of the magnitude and sign of the differential expression effect size for each gene. We start by translating each gene's *p* value into a chi-squared statistic

X_i with one degree of freedom. In its simplest form, the test is designed to assess whether genes are consistently over-expressed in a set of cases compared to controls. Therefore, the test statistic T is the sum over all genes of the X_i, if X_i is positive, or 0, if X_i is negative. The distribution of this statistic under the null is obtained by assuming that each gene has a 50% chance of being over-expressed and 50% of being under-expressed. Note that this can be refined by computing the genome-wide probability of over-expressed genes, in case that the proportion differs from 50%. With either of these assumptions, the probability of observing a number *K* of genes over-expressed among the *n* genes in the module can be computed using a binomial distribution. For a given *K*, the statistic T is chi-squared with *K* degrees of freedom (because it is the sum of *K* one degree of freedom chi-square distributions). The probability of observing a test statistic greater than the observed value T can then be computed using a weighted sum of probabilities, summing over all possible values of *K*. The derivation is analogous if all genes are expected to be under-expressed. If the expectation is a mixture of over and under expressed genes, the T statistic is then summed over all genes in a manner that reflects that combination.

Supplemental References

1. Henri S, Poulin LF, Tamoutounour S, Ardouin L, Guilliams M, de Bovis B, et al. CD207+ CD103+ dermal dendritic cells cross-present keratinocyte-derived antigens irrespective of the presence of Langerhans cells. *J Exp Med.* 2010;207(1):189-206.
2. Irizarry RA, Bolstad BM, Collin F, Cope LM, Hobbs B, and Speed TP. Summaries of Affymetrix GeneChip probe level data. *Nucleic Acids Res.* 2003;31(4):e15.
3. Irizarry RA, Hobbs B, Collin F, Beazer-Barclay YD, Antonellis KJ, Scherf U, et al. Exploration, normalization, and summaries of high density oligonucleotide array probe level data. *Biostatistics.* 2003;4(2):249-64.
4. Carvalho BS, and Irizarry RA. A framework for oligonucleotide microarray preprocessing. *Bioinformatics.* 2010;26(19):2363-7.
5. Leek JT, Johnson WE, Parker HS, Jaffe AE, and Storey JD. The sva package for removing batch effects and other unwanted variation in high-throughput experiments. *Bioinformatics.* 2012;28(6):882-3.
6. Reich M, Liefeld T, Gould J, Lerner J, Tamayo P, and Mesirov JP. GenePattern 2.0. *Nat Genet.* 2006;38(5):500-1.
7. Artyomov MN, Munk A, Gorvel L, Korenfeld D, Cella M, Tung T, et al. Modular expression analysis reveals functional conservation between human Langerhans cells and mouse cross-priming dendritic cells. *J Exp Med.* 2015;212(5):743-57.
8. Pan JB, Hu SC, Shi D, Cai MC, Li YB, Zou Q, et al. PaGenBase: a pattern gene database for the global and dynamic understanding of gene function. *PLoS One.* 2013;8(12):e80747.
9. Cock PJ, Fields CJ, Goto N, Heuer ML, and Rice PM. The Sanger FASTQ file format for sequences with quality scores, and the Solexa/Illumina FASTQ variants. *Nucleic Acids Res.* 2010;38(6):1767-71.
10. Suzuki R, and Shimodaira H. Pvclust: an R package for assessing the uncertainty in hierarchical clustering. *Bioinformatics.* 2006;22(12):1540-2.

11. Barbie DA, Tamayo P, Boehm JS, Kim SY, Moody SE, Dunn IF, et al. Systematic RNA interference reveals that oncogenic KRAS-driven cancers require TBK1. *Nature.* 2009;462(7269):108-12.
12. Hanzelmann S, Castelo R, and Guinney J. GSVA: gene set variation analysis for microarray and RNA-seq data. *BMC Bioinformatics.* 2013;14:7.
13. Best JA, Blair DA, Knell J, Yang E, Mayya V, Doedens A, et al. Transcriptional insights into the CD8(+) T cell response to infection and memory T cell formation. *Nat Immunol.* 2013;14(4):404-12.
14. Gartlan KH, Markey KA, Varelias A, Bunting MD, Koyama M, Kuns RD, et al. Tc17 cells are a proinflammatory, plastic lineage of pathogenic CD8+ T cells that induce GVHD without antileukemic effects. *Blood.* 2015;126(13):1609-20.
15. Ramesh R, Kozhaya L, McKevitt K, Djuretic IM, Carlson TJ, Quintero MA, et al. Pro-inflammatory human Th17 cells selectively express P-glycoprotein and are refractory to glucocorticoids. *J Exp Med.* 2014;211(1):89-104.
16. Langfelder P, and Horvath S. WGCNA: an R package for weighted correlation network analysis. *BMC Bioinformatics.* 2008;9:559.
17. Langfelder P, and Horvath S. Fast R Functions for Robust Correlations and Hierarchical Clustering. *J Stat Softw.* 2012;46(11).
18. Langfelder P, Luo R, Oldham MC, and Horvath S. Is my network module preserved and reproducible? *PLoS Comput Biol.* 2011;7(1):e1001057.
19. Shannon P, Markiel A, Ozier O, Baliga NS, Wang JT, Ramage D, et al. Cytoscape: a software environment for integrated models of biomolecular interaction networks. *Genome Res.* 2003;13(11):2498-504.
20. Janky R, Verfaillie A, Imrichova H, Van de Sande B, Standaert L, Christiaens V, et al. iRegulon: from a gene list to a gene regulatory network using large motif and track collections. *PLoS Comput Biol.* 2014;10(7):e1003731.

Supplemental Table 1, included in a separate Excel file. Characterization of the 19 highly preserved F→M WGCNA gene modules, and the 2 skin-specific B6→129 WGCNA modules: (**A**) over-represented GO categories, (**B**) putative driver genes identified on the basis of high intra-modular connectivity, and (**C**) transcription factors predicted to act as upstream regulators. AUC, area under the curve; FDR, false discovery rate; kME, module eigengene-based connectivity; NES, normalized enrichment score.

Supplemental Table 2, included in a separate Excel file. Clinical details concerning the 5 patients included in the paired-tissue RNAseq analysis. ALL, acute lymphoblastic leukemia; AML, acute myeloid leukemia; 2ry AML, secondary acute myeloid leukemia; CR1, first complete remission; CR2, second complete remission; DLI, donor lymphocyte infusion; Haplo, haploidentical donor; HL, Hodgkin s lymphoma; MMF, mycophenolate mofetil; MUD, matched unrelated donor; SIB, sibling donor; TBI, total body irradiation.

Supplemental Table 3, included in a separate Excel file. (A) Results from the pre-processing performed to test for LC contaminants in LC-replete versus LC-depleted epidermal samples. (**B**) List of the intestine- and skin-specific genes subtracted from the analysis. DT, diphtheria toxin; FDR, false discovery rate; SD, standard deviation.

Supplemental Table 4, included in a separate Excel file. Tc1, Tc17 and MDR1^{+} Th1/Th17 gene signatures.

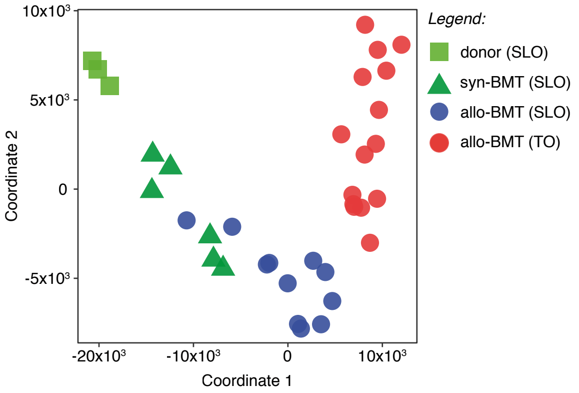

Supplemental Figure 1. MDS of SLO and TO-derived T_E samples in B6→129 BMT model: no prior subtraction of skin- and gut-derived gene sets. MDS plot showing the proximity of the complete transcriptional profiles of donor-derived $CD8^+$ T cells isolated from different organs.

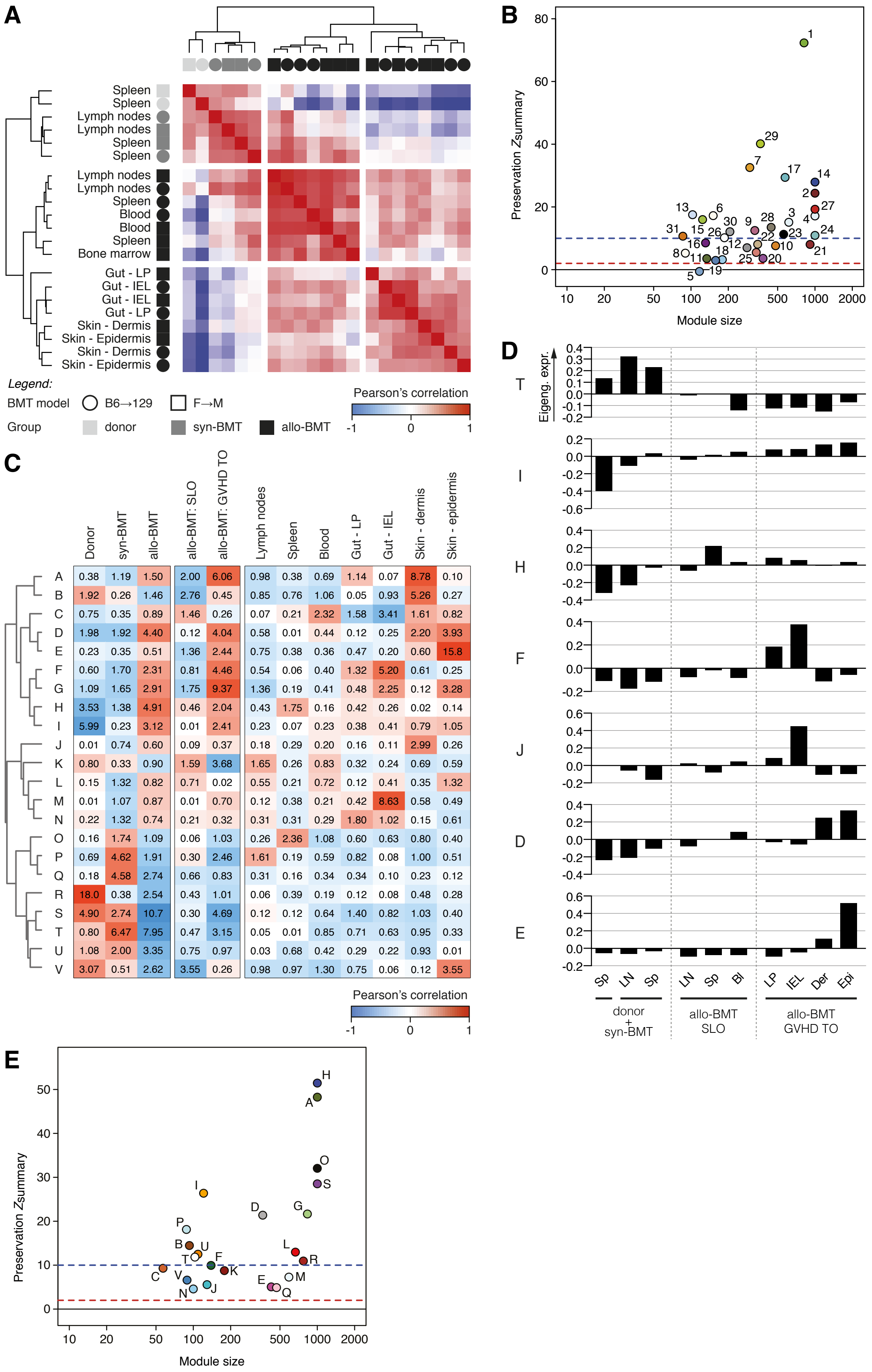

Supplemental Figure 2. Spatial diversity of T_E profiles is conserved between two independent experimental BMT models. (**A**) Average linkage hierarchical clustering of all samples from B6→129 and F→M BMT models and controls, linked to a similarity matrix with color coded Pearson's correlation coefficients to show clustering according to experimental system (circles: B6→129; squares: F→M) and sample cohort (light grey: donor; dark grey: syn-BMT; black: allo-BMT). (**B**) F→M BMT module preservation in B6→129 BMT model showing the summary statistics $Z_{summary}$ as a function of the module size (red line $Z_{summary}$ = 2; blue line $Z_{summary}$ = 10). (**C**) Correlation matrix depicting the association between the 22 WGCNA gene modules defined in B6→129 dataset (MA-MV) and the experimental groups (donor, syn-BMT, allo-BMT), GVHD subgroups (SLO, TO), and GVHD individual organs. Cell color and cell number indicate Pearson's correlation coefficient and corresponding -log10(*p*-value), respectively. (**D**) Bar graphs showing the mean eigengene expression in each of the tissues, for donor a syn-BMT (MT), pan-GVHD TO (MI), organ-selective (MH, MF, MD) and tissue-specific modules (MJ, ME). (**E**) B6→129 BMT module preservation in F→M BMT model showing the summary statistics $Z_{summary}$ as a function of the module size (red line $Z_{summary}$ = 2; blue line $Z_{summary}$ = 10).
Bl, Blood; Der, dermis; Epi, epidermis; IEL, intraepithelial lymphocytes; LN, lymph nodez; LP, lamina propria; SLO, secondary lymphoid organs; Sp, spleen; TO, target organs.

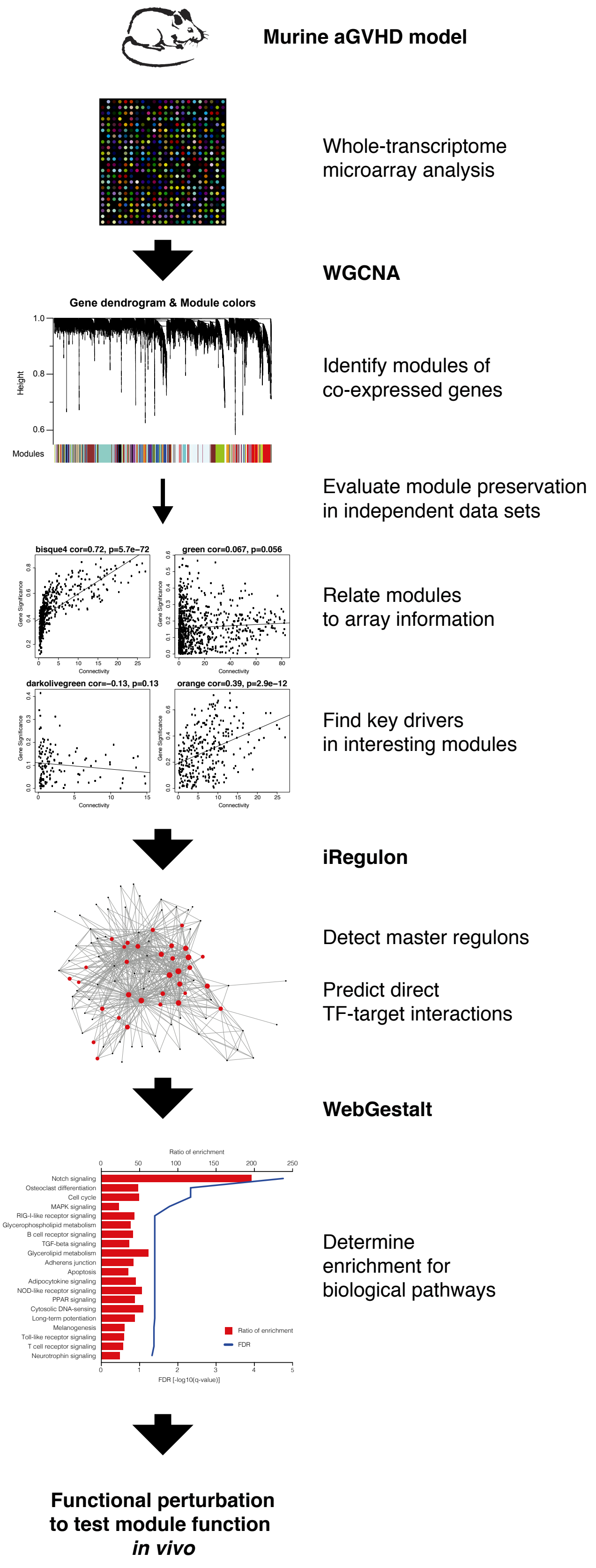

Supplemental Figure 3. Analytical pipeline. A summary of analytical process following WGCNA is shown. See Results text for details.

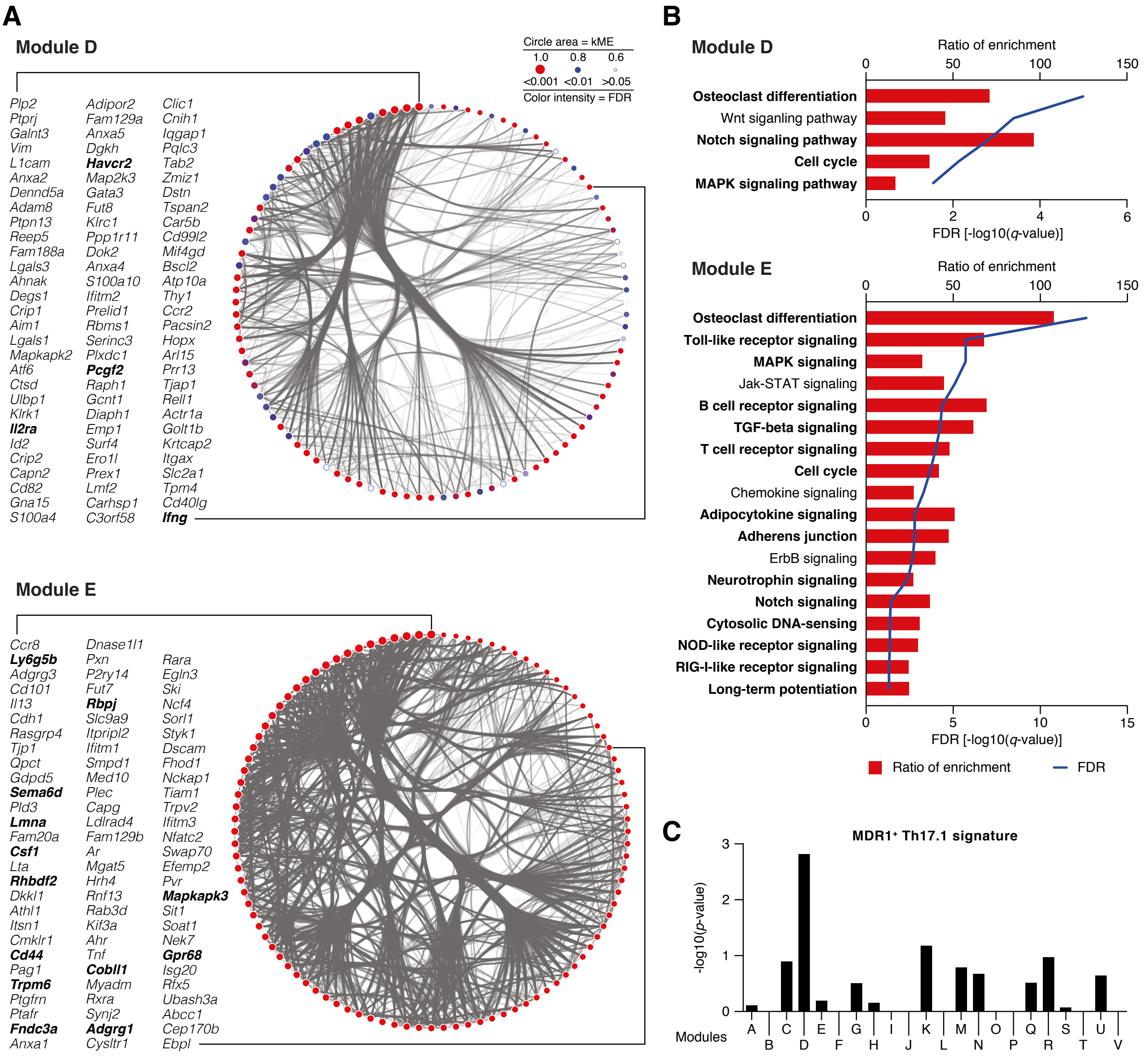

Supplemental Figure 4. Module M28 (defined in the F→M BMT model) overlaps with 2 epidermis-specific modules (MD and ME) independently identified in the B6→129 BMT model. (**A**) Cytoscape generated visualization of the network connections among the 100 most connected genes in B6→129 MD and ME. Nodes represent the genes (circle area proportional to the intra-modular connectivity, kME) and the color reflects the FDR *q*-value of its correlation with the module; edges represent the topological overlap between genes (line thickness proportional to adjacency). Driver genes common to F→M M28 are highlighted in bold. (**B**) Graphs showing the ratio of enrichment (bars) and FDR *q*-values (line) for KEGG pathways predicted by WebGestalt to regulate MD and ME. Pathways common to F→M M28 are highlighted in bold. (**C**) Graph showing module association with immunossupressive therapy resistance assessed by determining the over-representation of a gene signature specific for a human MDR1+ Th1/Th17 subset that is resistant to glucocorticoids. Hypergeometric test.
FDR, false discovery rate; kME, intra-modular connectivity.

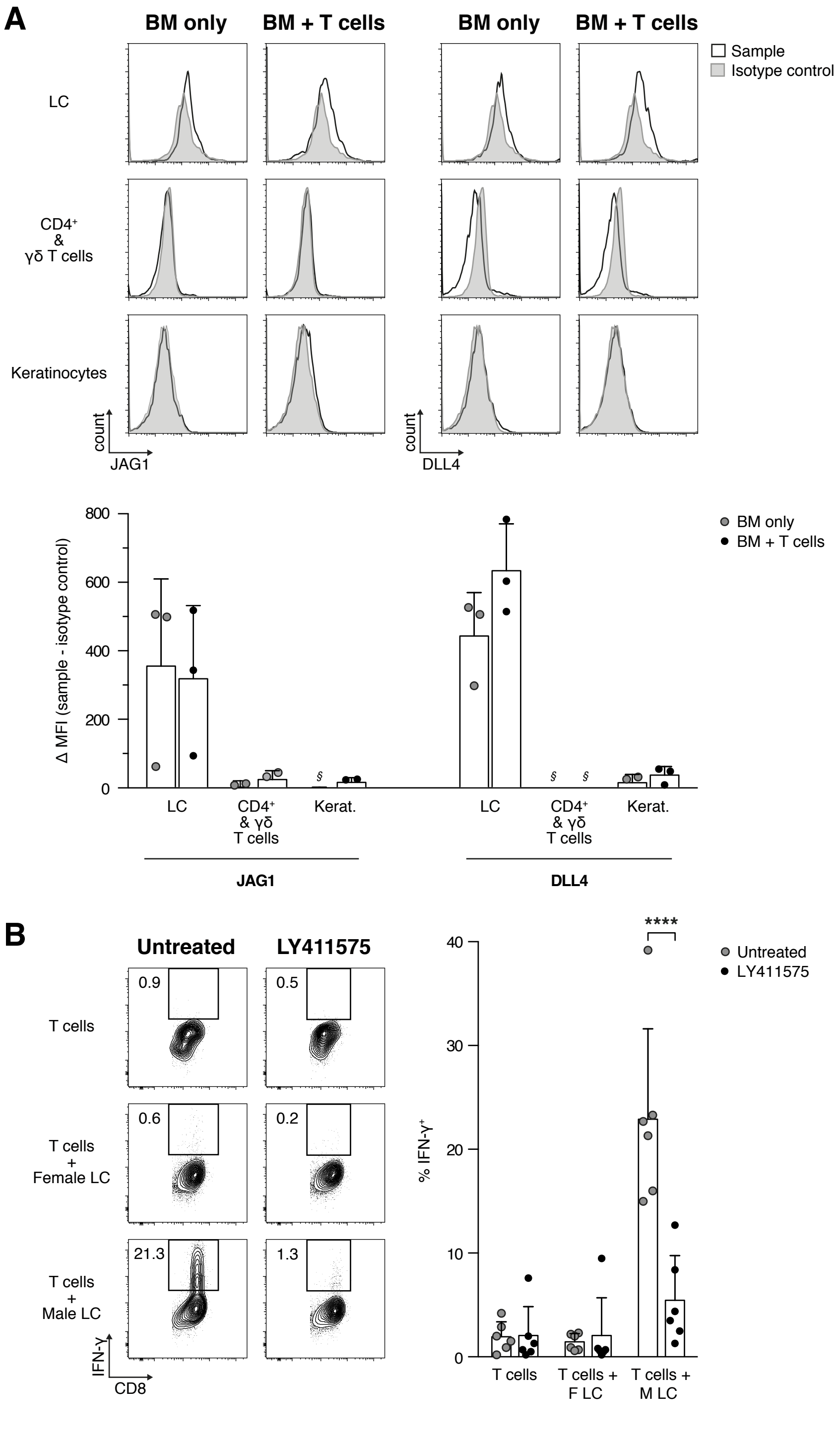

Supplemental Figure 5. LC express higher levels of JAG1 and DLL4 than other epidermal cell populations. (**A**) Notch ligand expression by the main cell populations in the epidermis post-BMT. *Top* - Representative flow cytometric plots showing JAG1 and DLL4 expression by LC versus $CD4^+$/γδ T cells versus keratinocytes in the absence (BM only) or presence (BMT + T cells) of GVHD. $CD8^+$ T cells infiltrating the epidermis in GVHD were excluded by gating. *Bottom* - Summary data of the difference in MFI staining between each sample and the respective isotype control (n = 2, graphs show mean ± SD; §, isotype control MFI > sample MFI). No detectable DLL1 and JAG2 expression was found on any epidermal population (data not shown). (**B**) Effect of in vitro LY411575 or PBS (untreated) exposure upon IFN-γ generation by concanalavin-preactivated MataHari T cells upon interaction with male or female LC. Data derived from 6 independent experiments, graphs show mean ± SD. ****$p \leq 0.0001$, two-way ANOVA with Holm-Sidak correction for multiple comparisons.
BM, bone marrow; F LC, female Langerhans cells; M LC, male Langerhans cells; MFI, median fluorescence intensity.

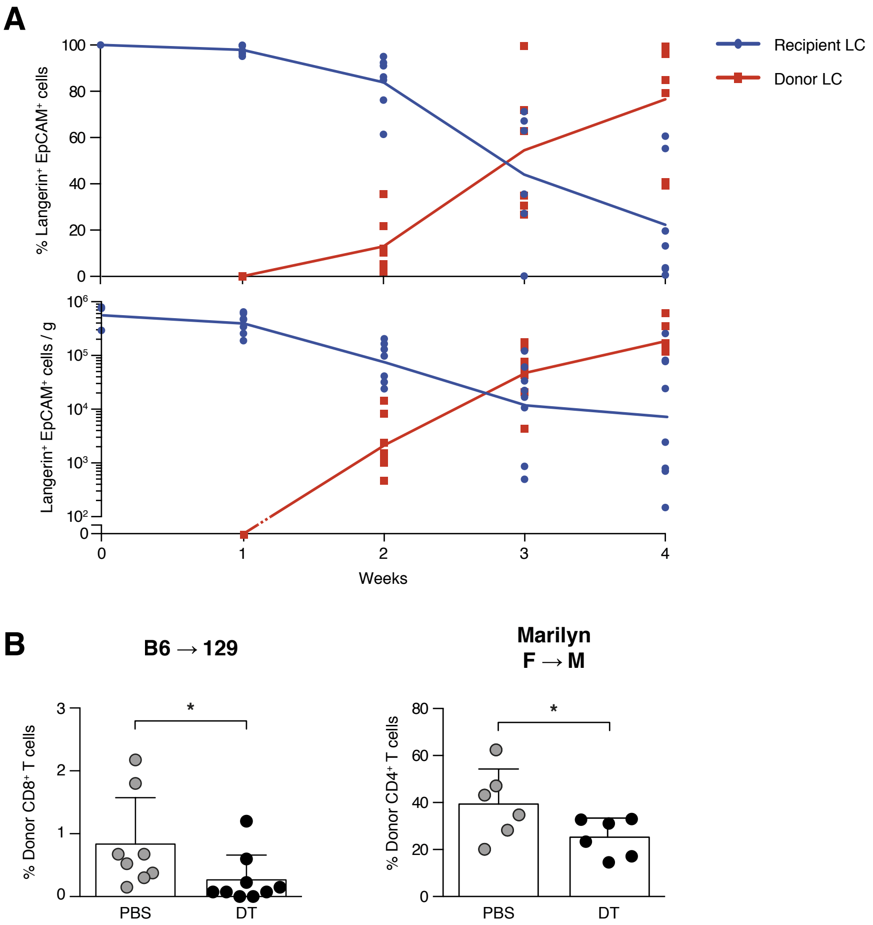

Supplemental Figure 6. Host-derived LC remain the dominant LC population in the epidermis in the first two weeks post-BMT and their presence is required for epidermal T_E accumulation. (**A**) Kinetics of replacement of host-derived LC by donor-derived LC after BMT. Graphs showing the relative proportion (top) and absolute number (bottom) of host- and donor-derived LC over the first 4 weeks post-transplant. Baseline (n = 3), week 1 (n = 7), week 2 (n = 7), week 3 (n = 8), week 4 (n = 8). Lines indicate mean (top graph) and geometric mean (bottom graph). (**B**) Effect of presence or absence of host LC, upon $CD8^+$ T_E accumulation in the epidermis in the B6→129 BMT model (left) and $CD4^+$ T_E Marilyn accumulation in the F→M BMT model (right). B6→129, n = 8-9/group; Marilyn F→M, n = 6/group. Bars indicate mean + SD. *$p \leq 0.05$, two-tailed Mann-Whitney test. LC, Langerhans cells.
